# Gut microbiota modulates CNS barrier function in murine model for multiple sclerosis

**DOI:** 10.1186/s12987-025-00724-y

**Published:** 2025-11-07

**Authors:** Junhua Xie, Marlies Burgelman, Pieter Dujardin, Jonas Castelein, Griet Van Imschoot, Elien Van Wonterghem, Hannah Lernout, Sjoerd T. T. Schetters, Charysse Vandendriessche, Lien Van Hoecke, Roosmarijn E. Vandenbroucke

**Affiliations:** 1 VIB-UGent Center for Inflammation Research, VIB, Ghent, 9052 Belgium; 2 Department of Biomedical Molecular Biology, Ghent University, Ghent, 9052 Belgium; 3 Department of Internal Medicine and Pediatrics, Ghent University, Ghent, 9000 Belgium

**Keywords:** Gut microbiota, Experimental autoimmune encephalomyelitis, Multiple sclerosis, Blood-brain barrier, Blood-cerebrospinal fluid barrier, Blood-spinal cord barrier, Short-chain fatty acids, Gut–brain axis

## Abstract

Multiple sclerosis (MS) is a chronic inflammatory disease that has been associated with dysfunction of the protective barriers in the central nervous system (CNS), allowing harmful immune cells to enter. Growing evidence implicates gut microbiota dysbiosis in MS pathogenesis, yet its connection to CNS barrier integrity is not fully understood. To address this, we investigated the role of gut microbiota in experimental autoimmune encephalomyelitis (EAE), a mouse model of MS, by depleting intestinal microbes using a broad-spectrum antibiotic (ABX) regimen. Our results indicate that ABX-mediated gut microbiota depletion was associated with a milder EAE disease course, accompanied by reduced immune cell infiltration and attenuation of EAE-associated loss of barrier integrity. These improvements coincided with preserved expression and localization of tight junction (TJ) proteins, vital for maintaining barrier integrity. While these data support a microbiota-mediated effect, ABX compounds may also exert microbiota-independent immunomodulatory actions. Importantly, although ABX treatment proved beneficial in the inflammatory EAE setting, antibiotics are also known to disrupt immune and barrier homeostasis under steady-state conditions, highlighting their dual and context-dependent nature. To further look into this, we transferred gut microbiota between EAE and healthy mice and observed a microbiota-dependent effect on CNS barrier integrity. Next, treatment with SCFAs partly restored the disrupted CNS barriers and was linked to reduced disease development in EAE. Altogether, our findings suggest that the gut microbiota has dual role in the CNS barriers in MS. These findings align with emerging literature identifying gut-derived signals, particularly under antibiotic perturbation or through microbial metabolites, as critical modulators of neuroimmune homeostasis. Additionally, our results highlight the potential of microbiota-targeted interventions and SCFAs to influence CNS barrier integrity and disease outcomes.

**Supplementary Information:**

The online version contains supplementary material available at 10.1186/s12987-025-00724-y.

## Introduction

Multiple sclerosis (MS) stands as a chronic inflammatory disease of the central nervous system (CNS), marked by the invasion of autoreactive immune cells that mediate demyelination of CNS axons [1–3]. Throughout the course of the disease, the entry of immune cells into the CNS might be aided by augmented permeability and disruption of the CNS barriers, including the blood-brain barrier (BBB), the meninges, the blood-spinal cord barrier and the blood-cerebrospinal fluid (CSF) barrier [4–6]. These barriers play a crucial role in preserving CNS homeostasis by regulating the exchange of substances between the blood and the CNS [7–9]. Consequently, dysfunction in CNS barriers is widely believed to contribute significantly to the development and progression of MS [10]. Therefore, gaining a comprehensive understanding of the underlying mechanisms driving CNS barrier dysfunction in MS is paramount for the advancement of effective therapeutic interventions to combat this debilitating disease.

Emerging research has shed light on the influence of the gut microbiome and gut-brain axis signaling in modulating the permeability and development of CNS barriers. Although the precise mechanisms linking the gut microbiota to CNS barrier regulation are not yet fully understood. Under homeostatic conditions, studies using broad-spectrum antibiotics (ABX) and/or germ-free (GF) mice have shown increased permeability of the BBB and blood–CSF barrier, accompanied by altered expression of tight junction (TJ) proteins [11–14]. Among these, occludin has emerged as a particularly sensitive marker of CNS barrier integrity, with reduced expression in CNS microvessels correlating with barrier leakiness and immune cell infiltration under homeostasis [15, 16]. By contrast, the consequences of microbiota depletion in inflammatory settings, and specifically in experimental autoimmune encephalomyelitis (EAE), are less well defined and may diverge from those observed under steady-state conditions.

A mounting body of evidence highlights the presence of disruptions in the microbiome and gut-brain axis in MS patients [17]. Indeed, dysbiosis has been observed in MS patients, with relapsing-remitting (RR) MS patients with active disease exhibit a trend to a less diverse microbiome compared to healthy controls [18, 19]. Similar alterations are reported in a mouse model of MS, where microbiota composition shifts during EAE progression [20]. Interestingly, ABX treatment in EAE models has been found to ameliorate disease severity, likely by modulating gut microbiota composition and dampening pro-inflammatory immune responses [21]. Specific microbes, such as segmented filamentous bacteria, promote Th17 differentiation and thereby contribute to CNS autoimmunity [22, 23]. The extent of ABX-mediated protection depends on the type, timing, and dose of treatment, and may reflect both reduced bacterial load and changes in microbial composition. In addition, certain ABX (e.g., fluoroquinolones) exert direct immunomodulatory effects independent of microbiota alterations. Conversely, early-life ABX exposure has been linked to worsened EAE severity, underscoring the importance of treatment context and microbial resilience [24]. Finally, microbiota-driven changes in brain physiology may occur not only through immune modulation but also via non-immune mechanisms, including vagal and sympathetic signaling and microbial-derived metabolites [25].

Among these metabolites, short-chain fatty acids (SCFAs), the major fermentation products of dietary fibers and resistant starch, have been shown to support BBB and blood–CSF barrier integrity by enhancing TJ protein expression and localization in both in vitro and in vivo settings [11, 14, 26, 27]. SCFAs may influence barrier function either directly, via systemic circulation, or indirectly by activating free fatty acid receptors (FFAR2/3) expressed on endothelial cells [28, 29]. Importantly, both MS patients and EAE models exhibit microbial dysbiosis and dysregulated levels of SCFA in serum and stool, which correlate with disease activity and barrier dysfunction [30–34].

Although perturbations at both the level of the gut microbiota and the CNS barriers have been described in MS, the link between the gut microbiota and CNS barriers in MS remains poorly understood. In this study, we further investigated how gut microbiota composition influences CNS barrier integrity in EAE. Our data indicate that EAE-associated microbiota correlates with altered TJ expression and compromised barrier function. Moreover, microbiota transfer from healthy donors and SCFA supplementation were associated with partial preservation of CNS barrier integrity and reduced EAE severity.

## Materials and methods

### Animals

Female C57BL/6J mice (Janvier) were housed in a specific pathogen-free (SPF) animal facility, in groups of 4–6 per cage with *ad libitum* access to food and water and a 14 h light/10 h dark cycle. All experiments were officially approved by the ethical committee of the Faculty of Sciences, Ghent University (EC2022-059, EC2023-018).

### EAE induction

EAE was induced in 10 weeks old, female C57BL/6J mice by subcutaneous injection of 200 µg MOG_(35−55)_ peptide, emulsified with complete Freund’s adjuvant emulsion (EK-2110; Hooke Laboratories). Immediately after MOG injection, pertussis toxin (PTX; EK-2110; Hooke Laboratories) was administered intraperitoneally (~ 120 ng/mouse). Additionally, a second PTX boost injection was administered 24 h after the first PTX injection. All injections were performed under anesthesia with 2% isoflurane. From day 7 post-immunization (p.i.) onwards, the body weight of the mice as well as their disease score was evaluated on a daily basis. Disease scores were given according to the following guidelines: 0, no clinical disease; 1, weakness of tail; 2, complete tail paralysis; 3, partial hind limb paralysis; 4, complete hind limb paralysis; 5, moribund or dead.

### Depletion of the gut microbiota

To deplete the gut microbiota, the mice were provided with drinking water containing 0,2 g/L ciprofloxacin (17850; Sigma), 1 g/L ampicillin (A9518; Sigma), 0,5 g/L vancomycin (V0155.0005; Duchefa Biochemie) and 1 g/L metronidazole (M1547; Sigma) as described previously [14]. The ABX-supplemented drinking water was refreshed every 3 days. ABX treatment was started 2 weeks before EAE induction and continued until the end of the experiment. Control groups received normal drinking water.

### Flow cytometry based immunophenotyping of blood

Blood was collected in EDTA coated tubes followed by an ACK lysis step (Westburg) for 2 min at RT. Next, the cells were incubated for 30 min at 4 °C with Ab-mix containing Fc-block, viability dye, anti-CD45, anti-γδTCR, anti-Ly6C, anti-MHCII, anti-Siglec F, anti-CD90, anti-Ly6G, anti-CD4, anti-ST2, anti-CD43, anti-CXCR3, anti-CD62L, anti-CD8, anti-CD11c, anti-CD3, anti-CD19, anti-CD200R3, anti-CD11b and anti-NK1.1 Flow cytometry data were analyzed first using FlowJo analysis software. First, files were compensated using UltraComp eBeads (Thermo Fisher) microspheres labeled with the appropriate antibodies. Compensation was additionally verified using fluorescence-minus-one (FMO) controls for every single fluorochrome. Next, gating was performed on a stable flow (time vs. cell count), subsequently on viability dye-negative/CD45-positive cells and finally on single cells (FSC-A/FSC-H). Using the ViSNE module, we generated tSNE plots. Cells were clustered by γδTCR, Ly6C, MHCII, Siglec F, CD90, Ly6G, CD4, ST2, CD43, CXCR3, CD62L, CD8, CD11c, CD3, CD19, CD200R3, CD11b and NK1.1 expression. Next, we identified and manually gated subpopulations, as represented by the tSNE clustering analysis, to generate the subpopulations as represented in the graphs. After defining gating strategies, the individual experimental samples were similarly gated in FlowJo and statistics were exported to GraphPad Prism 7 for visualization.

### Fecal microbiome transplant (FMT)

Donor mice were euthanized in a CO_2_ chamber and the fecal content of the cecum was immediately isolated and homogenized in D-PBS. The fecal homogenate was directly transferred to the recipients by oral gavage (200 µl/mouse). FMT recipient mice were each time gavaged with freshly homogenized fecal material for 5 consecutive days. Pre-treatment with ABX was deliberately avoided, as ABX-induced barrier weakening could confound subsequent analyses of CNS integrity [35]. Instead, we applied repeated daily gavages to promote engraftment. Donor feces were pooled across at least two cages to minimize cage-specific effects. Efficiency of engraftment was verified by qPCR, which revealed a consistent shift in selected bacterial taxa following healthy donor FMT (see Figure S6), indicating partial but detectable transfer of donor microbiota into the recipient mice. Six days after the last FMT, the recipient mice were sampled. When FMT recipients were EAE mice, FMT was performed at day 6 p.i. until day 10 p.i. For EAE mice that were used as FMT donors, sacrifice for feces donation was done at a clinical score of 2 or higher. A schematic timeline overview of the FMT experiments is provided in Figs. 3 and 4.

### Real-time PCR quantification of bacterial taxa

Fecal DNA was isolated using the QiaAmp PowerFecal Pro DNA Kit (Qiagen, 51804). The abundance of Alpha-Proteobacteria, Gamma-Proteobacteria, Bacteroidetes, Firmicutes, and Actinobacteria was determined using qPCR. Universal primers were used to determine the relative abundance. Primer sequences are displayed in Table 1.

**Table 1:** Overview of the primers used to determine the abundance of Alpha-Proteobacteria, Gamma-Proteobacteria, Bacteroidetes, Firmicutes, and actinobacteria

	Forward	Reverse
Alpha-Proteobacteria	CIAGTGTAGAGGTGAAATT	CCCCGTCAATTCCTTTGAGTT
Gamma-Proteobacteria	TCGTCAGCTCGTGTYGTGA	CGTAAGGGCCATGATG
Bacteroidetes	CRAACAGGATTAGATACCCT	GGTAAGGTTCCTCGCGTAT
Firmicutes	TGAAACTYAAAGGAATTGACG	ACCATGCACCACCTGTC
Actinobacteria	TACGGCCGCAAGGCTA	TCRTCCCCACCTTCCTCCG
Universal	AAACTCAAAKGAATTGACGG	CTCACRRCACGAGCTGAC

### SCFA treatment

Mice were treated with SCFAs according to our previous protocol [14]. Specifically, sodium butyrate (303410; Merck) and sodium propionate (P1880; Merck) were dissolved in D-PBS and administered to EAE mice via oral gavage (1 g/kg body weight), starting at day 10 p.i. until day 15 p.i. One day after the last treatment (day 16 p.i.), the mice were sampled. Control EAE mice received oral gavages of D-PBS according to the same regime as the SCFA-treated EAE mice. Additionally, D-PBS-orally gavaged non EAE mice were included as healthy controls.

### Fecal metabolomics

One fecal pellet per mouse was collected, snap frozen and stored at -80 °C until analysis. Organic extraction of fecal metabolites was conducted using the Ribolyzer homogenizer. Five hundred µl of extraction buffer (80% MeOH) was added to the feces and insolubilities were removed by centrifugation (20,000 g, 15 min at 4 °C). MS method Mass Spectrometry measurements were performed using Dionex UltiMate 3000 LC System (Thermo Scientific) coupled to a Q Exactive Orbitrap mass spectrometer (Thermo Scientific) operated in negative mode. Ten µl of sample was injected onto a Poroshell 120 HILIC-Z PEEK Column (Agilent InfinityLab). A linear gradient was carried out starting with 90% solvent A (acetonitrile) and 10% solvent B (10 mM Na-acetate in mqH_2_O, pH 9.3). From 2 to 12 min the gradient changed to 60% B. The gradient was kept on 60% B for 3 min and followed by a decrease to 10% B. The chromatography was stopped at 25 min and the flow was kept constant at 0.25 ml/min. The columns temperature was kept constant at 25 °C. The mass spectrometer operated in full scan (range [70.0000-1050.0000]) and negative mode using a spray voltage of 2.8 kV, capillary temperature of 320 °C, sheath gas at 45, auxiliary gas at 10. AGC target was set at 3.0E + 006 using a resolution of 70,000. Data collection was performed using the Xcalibur software (Thermo Scientific). The data analyses were performed by integrating the peak areas (El-Maven - Polly - Elucidata).

### Quantitative assessment of barrier leakage

Mice were i.v. injected with 70 kDa (D1957; ThermoFisher Scientific) or 3 kDa (D7135; ThermoFisher Scientific) fixable biotinylated dextran (BD). We used 70 kDa BD in the ABX experiments, where barrier disruption was expected to be more pronounced, in order to capture gross leakage. In the FMT and SCFA experiments, where subtler differences in barrier integrity were anticipated, we used the smaller 3 kDa BD tracer to increase sensitivity to mild tight junction alterations. Fifteen minutes after i.v. injection, mice were anesthetized with ketamine/xylazine (100 mg/kg ketamine; 20 mg/kg xylazine in D-PBS). The brain was dissected from the skull whereas the spinal cord was isolated via hydraulic extrusion. Tissues were fixed O/N in 4% PFA at 4 °C. To prepare for cryo-preservation, samples were sequentially incubated in 15% and 30% sucrose (27,483,294; VWR) in D-PBS solutions at 4 °C. Next, tissue samples were embedded in NEG-50 (6502; Prosan bvba) and stored at -80 °C.

For staining, samples were cut into 20 μm cryosections (CryoStar NX70, Thermo Scientific). Next, sections were permeabilized in 0.3% PBST (PBS containing 0.3% Triton X-100), followed by blocking with goat immunomix (GIM) (5% goat serum in 0.3% PBST) at RT for 1 h, and incubation with anti-CD31 (553370, BD Pharmingen; 1:100) in GIM at 4 °C O/N. After washing with PBS, sections were stained with fluorophore-conjugated or streptavidin fluorophore-conjugated secondary antibodies in 0.1% PBST at RT for 1.5 h. Next, samples were counterstained with DAPI reagent (Sigma-Aldrich, 1:1000 in PBS) and the sections were mounted. For BD quantification, the images from hippocampus, choroid plexus (ChP) and the lumbar spinal cord were first processed with Image J and the intravascular BD signal was removed in Adobe Photoshop CS6 (i.e., BD signal that co-localizes with the CD31). The extravascular BD signal was measured in Image J as barrier leakage properties.

### Immunostaining

Mice were sedated with ketamine/xylazine (100 mg/kg ketamine; 20 mg/kg xylazine in D-PBS), followed by transcardial perfusion with 0.2% heparin in D-PBS (2.5 ml/min, 10 ml/mouse). Next, the brain was isolated and fixed overnight in 4% PFA at 4 °C. Spinal cord was collected via hydraulic extrusion and fixed overnight in 4% PFA at 4 °C.

After fixation, tissue samples were dehydrated and paraffinized. Paraffin-embedded brains and spinal cords samples were cut into 5 μm sections (HM 340 E, Thermo Scientific). For immunofluorescence staining, sections were permeabilized in 0.3% PBST (PBS containing 0.3% Triton X-100). Following blockage with goat immunomix (GIM) (5% goat serum in 0.3% PBST) at RT for 1 h, sections were incubated with primary antibodies in GIM at 4 °C overnight. After washing with PBS, sections were stained with fluorophore-conjugated secondary antibodies in 0.1% PBST at RT for 1 h. Next, samples were counterstained with DAPI reagent (Sigma-Aldrich, 1:1000 in PBS) and the sections were mounted. The following primary antibodies were used: anti-occludin (cat. no. 33-1500, Thermo Scientific; 1:100); anti-E-cadherin (cat. no. 610181, BD Biosciences; 1:500); anti-IBA1 (cat. no. 019-19741, Wako; 1:500); anti-CD3 (cat. no. A0452, Agilent; 1:200); and anti-CD31 (cat. no. DIA-310, Dianova; 1:100). For all immunostainings, lumbarspinal cord, hippocampus and ChP were evaluated in all experiments. The imaging was done with the 25×/40× oil objective using confocal microscopy (LSM780, Zeiss). All images were processed using Image J. For TJ quantification, the continuous TJ length was quantified with the Ridge Detection plugin for Image J. The percentage of TJ expression area was quantified by dividing the area of TJ signal by the area of epithelial nuclei in ChP or of blood vessel in spinal cord and hippocampus.

### Statistical analysis

Statistical significance was determined using unpaired Student’s t tests or Mann-Whitney tests to compare two groups or, for multiple comparison analysis, two-way ANOVA followed by Šidák post hoc test (GraphPad Prism 9). *p* < 0.05 was considered statistically significant, with * *p* < 0.05, ** *p* < 0.01, *** *p* < 0.001, **** *p* < 0.0001. Values were expressed as means ± SEM.

## Results

### The severity of EAE is reduced following ABX-mediated microbiota depletion

**Fig. 1:**
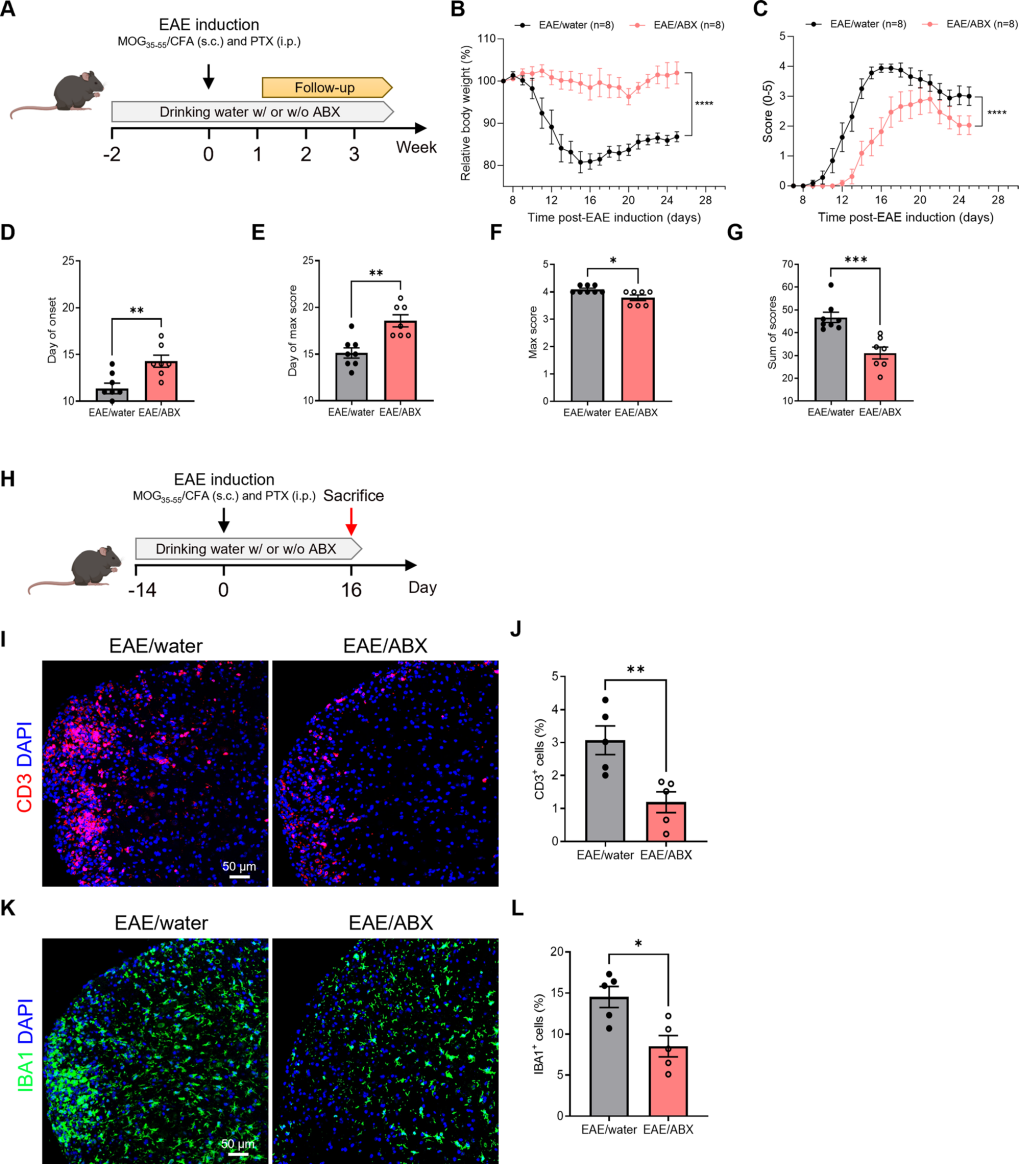
Antibiotics (ABX) mediated depletion of gut microbiota reduces the severity of EAE disease. (**A**) Schematic overview of the experimental set-up. ABX was added to the drinking water of the mice starting 2 weeks before EAE induction and was continued until the end of the experiment while control EAE mice received normal drinking water throughout the experiment. From day 7 post-immunization (p.i.) onwards, EAE mice were scored daily and their body weight was followed until day 25 p.i. (**B**-**C**) Relative body weight (BW, %) (**B**) and clinical scores (**C**) of ABX-treated EAE mice *versus* control EAE mice (*n* = 8). (**D**-**G**) Day of EAE onset (**D**), day of maximal score (**E**), maximal scores (**F**) and sum of all scores (**G**) measured over the whole follow-up period (until day 25 p.i.) (*n* = 7–8). (**H**) Schematic overview of the experimental set-up for sample isolation. (**I**) Representative images of spinal cord sections stained for CD3. Scale bar 50 μm. (**J**) Amount of CD3^+^ cells (%) relative to the amount of DAPI^+^ cells in spinal cord tissue (*n* = 5). (**K**) Representative images of spinal cord sections stained for IBA1. Scale bar 50 μm. (**L**) Amount of IBA1^+^ cells (%) relative to the amount of DAPI^+^ cells in spinal cord sections (*n* = 5). Datapoints represent mean ± SEM. Statistics for relative BW and scoring graphs (**B**-**C**) were conducted with a repeated measurements 2-way ANOVA test, comparing ABX- and control EAE mice. Statistical analysis for data linked to graphs D-G, J and L was performed with unpaired t-test or Mann-Whitney test. * *p* < 0.05; ** *p* < 0.01; *** *p* < 0.001; **** *p* < 0.0001. ABX, antibiotics; BD, Biotinylated dextran; BW, body weight; CFA, complete Freund’s adjuvant; EAE, experimental autoimmune encephalomyelitis; MOG, myelin oligodendrocyte glycoprotein; p.i., post-immunization; PTX, pertussis toxin

To investigate the impact of the gut microbiota on EAE disease course, we compared EAE mice receiving ABX in their drinking water with EAE mice receiving normal drinking water (Fig. 1A). As depicted in Fig. 1B, ABX-treated EAE mice showed limited body weight loss compared to control mice. Moreover, the overall EAE disease course of ABX-treated EAE mice was less severe (Fig. 1C). The impact on disease severity can mainly be attributed to a significant delay in the onset of EAE symptoms (Fig. 1D). Furthermore, the day of maximal scoring was significantly postponed in the case of ABX-mediated microbiome depletion (Fig. 1E). Although there was only a slight decrease in maximum scores (Fig. 1F), the cumulative clinical scores over the entire follow-up period shows a significant protection in ABX-treated EAE mice compared to EAE controls (Fig. 1G).

Next, we assessed whether this attenuated disease severity was associated with reduced immune cell infiltration and T-cell numbers were quantified in spinal cord sections of ABX-treated and control EAE mice at day 16 p.i. (Fig. 1H). As shown in Fig. 1I-J, CD3^+^ T-cell infiltration in the spinal cord was significantly decreased in ABX-treated compared to EAE control mice. In addition, also the percentage of IBA1^+^ cells as a measure for the amount of microglia and infiltrated peripheral macrophages, was significantly reduced in spinal cord sections of ABX-treated EAE mice (Fig. 1K-L) and decreased CD3^+^ T-cell (not significant trend) and IBA1^+^ cell infiltration was observed in the brain parenchyma (Figure S1A-D). Moreover, the number of CD3^+^ T-cells in the spinal cord, along with the count of IBA1^+^ cells in both the spinal cord and brain parenchyma, exhibited a significant negative correlation with the OCLN expressed area at the corresponding regions (Figure S2). Taken together, these results suggest that removing the gut microbiota by ABX treatment is associated with an attenuated EAE disease course and reduced immune cell infiltration, although whether barrier disruption precedes or follows immune entry cannot be concluded from our data.

Notably, a thorough immunophenotyping analysis of blood samples from mice receiving ABX in their drinking water for two weeks and those receiving regular water showed no significant differences in any of the identified immune cell subsets (Figure S3).

### Gut microbiota depletion attenuates OCLN alterations at the CNS barriers in EAE

**Fig. 2:**
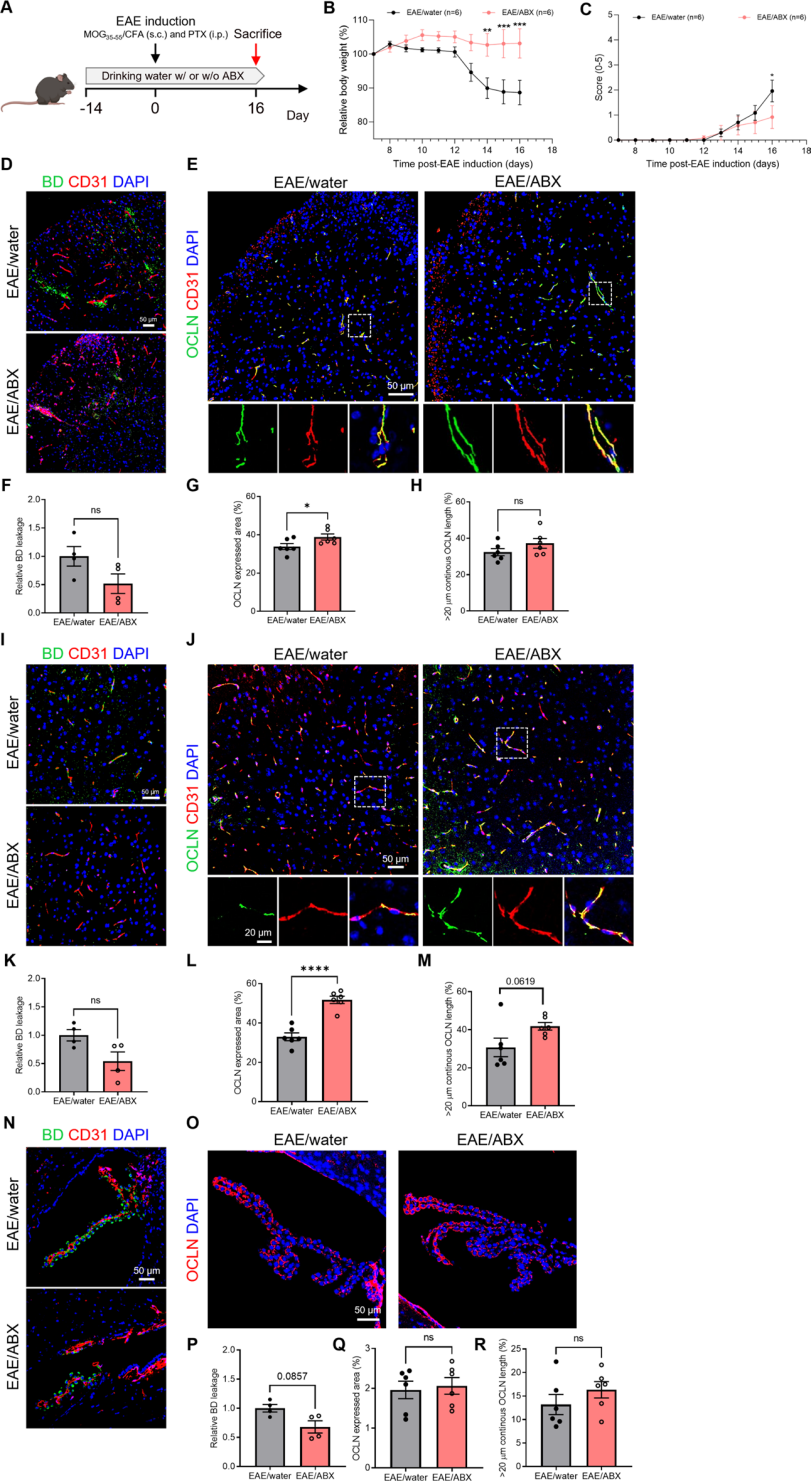
Depletion of gut microbiota partially prevents the EAE-induced CNS barrier disruption. (**A**) Schematic overview of the experimental set-up. ABX treatment was started 2 weeks before EAE induction and EAE mice were sampled at day 16 p.i. (**B**-**C**) Relative body weight (BW, %) (**B**) and clinical scores (**C**) of ABX-treated EAE mice *versus* control EAE mice (*n* = 6). (**D**-**E**) Representative images of immunohistochemical stainings for 70 kDa BD/CD31 (**D**) and OCLN/CD31 (**E**) on spinal cord sections. Scale bar 50 μm (**D**), 50 μm (up) and 20 μm (down) (**E**). (**F**-**H**) Quantification BD staining (**F**), OCLN expressed area (**G**) and OCLN continuous length of > 20 μm (**H**) in spinal cord tissue (*n* = 4–6; one section per mouse). (**I**-**J**) Representative images for 70 kDa BD/CD31 (**I**) and OCLN/CD31 (**J**) stainings of BBB blood vessels in the cortex. Scale bar 50 μm (**I**), 50 μm (up) and 20 μm (down) (**J**). (**K**-**M**) Quantification BD staining (**K**), OCLN expressed area (**L**) and OCLN continuous length of > 20 μm (M) in BBB blood vessels in the cortex (*n* = 4–6; one section per mouse). (**N**-**O**) Representative images for 70 kDa BD/CD31 (**N**) and OCLN (**O**) stainings on ChP. Scale bar 50 μm. (**P**-**R**) Quantification BD staining (**P**), OCLN expressed area (**Q**) and OCLN continuous length of > 20 μm (**R**) in ChP (*n* = 4–6; one section per mouse). Datapoints represent mean ± SEM. Statistical analysis was performed with unpaired t-test or Mann-Whitney test. * *p* < 0.05; ** *p* < 0.01; *** *p* < 0.001; **** *p* < 0.0001. ABX, antibiotics; BBB, blood-brain barrier; BD, biotinylated dextran; ChP, choroid plexus; CFA, complete Freund’s adjuvant; CNS, central nervous system; EAE, experimental autoimmune encephalomyelitis; MOG, myelin oligodendrocyte glycoprotein; OCLN, Occludin; PTX, pertussis toxin; TJs, tight junctions

CNS barrier dysfunction has been recognized as an early pathological feature and contributor to MS [36, 37] and gut microbiota dysbiosis is present in patients with MS^18, 19^. To investigate the role of the gut microbiome in influencing EAE-associated CNS barrier function, EAE mice were either treated with ABX or received normal drinking water and were sampled at day 16 p.i. (Fig. 2A). In Fig. 2B, it is illustrated that EAE mice treated with ABX experienced minimal body weight reduction compared to the control group. This difference is statistically significant from day 14 post-infection onward. Additionally, the progression of EAE disease in ABX-treated mice was milder, evidenced by significantly lower disease scores starting from day 16 post-infection, as shown in Fig. 2C. The leakage of 70 kDa biotinylated-dextran (BD) and the expression and subcellular localization of TJ proteins at the blood-spinal cord barrier, BBB, and blood-CSF barrier were analyzed. In the spinal cord, we observed a notable reduction in BD leakage upon ABX-mediated microbiome depletion in EAE mice (Fig. 2D and F). Furthermore, the expression of OCLN was significantly increased, while the OCLN continuous length was only slightly increased in ABX-treated compared non-ABX-treated EAE mice (Fig. 2E and G-H). Similar effects were observed for the BBB, including a decreased trend for BD leakage, significantly increased OCLN expression, and a trend for elevated OCLN continuous length (Fig. 2I-M). The effects of ABX-mediated microbiome depletion on the blood-CSF barrier, including barrier leakage and barrier integrity, were limited, with only a slight decrease in BD leakage observed, no alteration in OCLN expression and a slightly increased OCLN continuous length (Fig. 2N-R). Additionally, we analyzed CDH1 expression for the blood-CSF barrier, but this showed no significant differences (Figure S4). Overall, these findings indicate that ABX-mediated microbiome depletion positively affects the composition of OCLN-containing TJs at the CNS barrier in EAE disease, primarily at the blood-spinal cord barrier and BBB and to a more limited extend at the blood-CSF barrier.

### Transferring gut microbiota from EAE mice via fecal microbiota transfer (FMT) to healthy mice is associated with CNS barrier effects

**Fig. 3:**
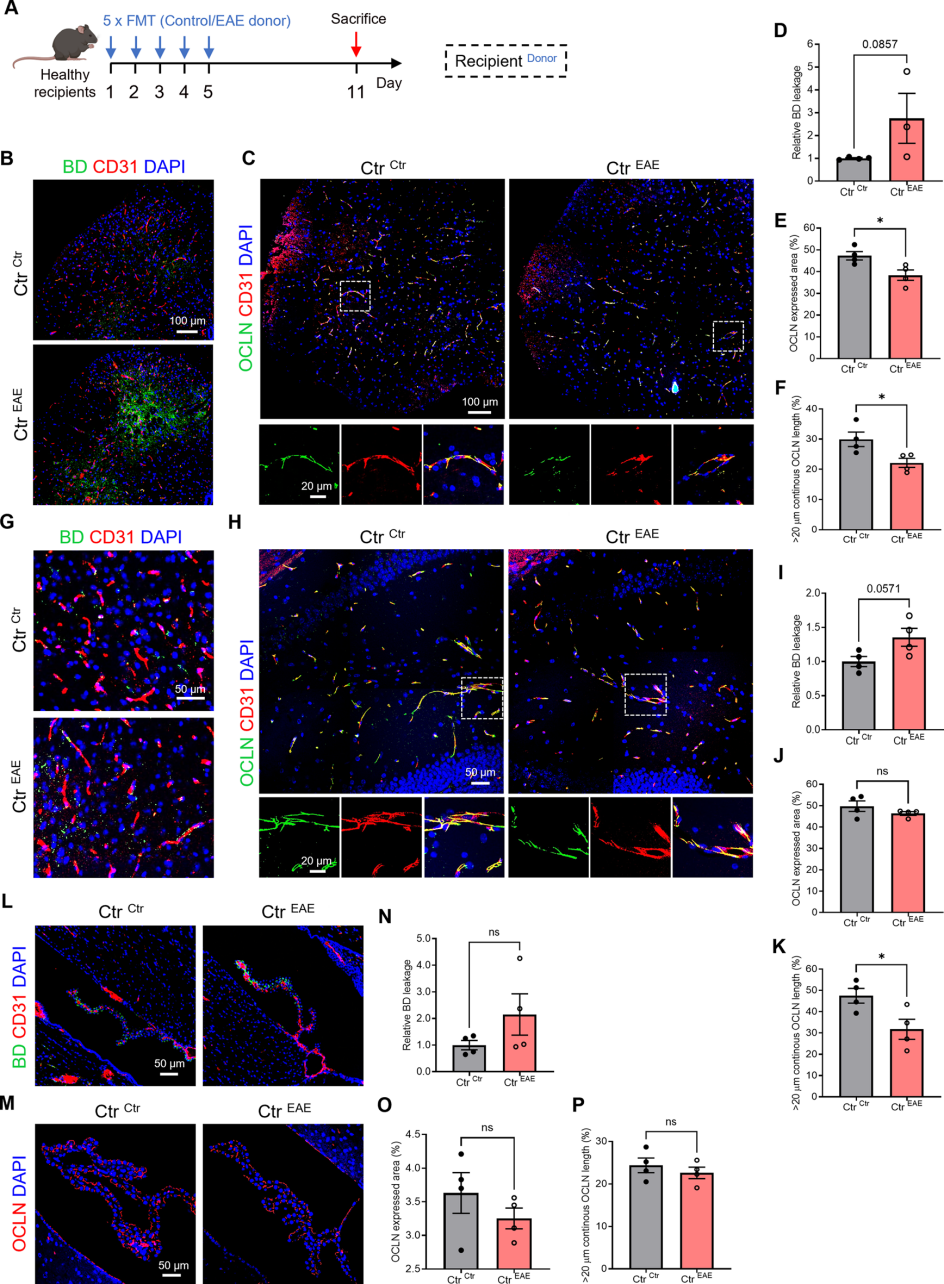
Gut microbiota from EAE mice impair CNS barrier integrity in healthy control mice. (**A**) Schematic overview of the experimental set-up. Healthy control (Ctr) mice were administered fecal contents from EAE mice or healthy controls orally for 5 consecutive days, and mice were sampled on day 11. (**B**-**C**) Representative images of immunohistochemical stainings for 3 kDa BD/CD31 (**B**) and OCLN/CD31 (**C**) on spinal cord sections. Scale bar 100 μm (**B**), 100 μm (up) and 20 μm (down) (**C**). (**D**-**F**) Quantification BD staining (**D**), OCLN expressed area (**E**) and OCLN continuous length of > 20 μm (**F**) in spinal cord tissue (*n* = 3–4). (**G**-**H**) Representative images for 3 kDa BD/CD31 (**G**) and OCLN/CD31 (**H**) stainings of BBB blood vessels in the cortex. Scale bar 50 μm (G), 50 μm (up) and 20 μm (down) (**H**). (**I**-**K**) Quantification BD staining (**I**) OCLN expressed area (**J**) and OCLN continuous length of > 20 μm (**K**) in BBB blood vessels in the cortex (*n* = 4). (**L**-**M**) Representative images for 3 kDa BD/CD31 (**L**) and OCLN (**M**) stainings on ChP. Scale bar 50 μm. (**N**-**P**) Quantification BD staining (**N**), OCLN expressed area (**O**) and OCLN continuous length of > 20 μm (**P**) in ChP (*n* = 4). Datapoints represent mean ± SEM. Statistical analysis was performed with unpaired t-test or Mann-Whitney test. * *p* < 0.05; ** *p* < 0.01; *** *p* < 0.001; **** *p* < 0.0001. BBB, blood-brain barrier; BD, biotinylated dextran; ChP, choroid plexus; CNS, central nervous system; EAE, experimental autoimmune encephalomyelitis; FMT, fecal microbiota transplantation; Ctr, healthy control; OCLN, Occludin; TJs, tight junctions

Based on our earlier findings and supported by existing literature indicating changes in gut microbiota during EAE onset [20], we delved into whether microbiota from EAE mice could affect CNS barrier integrity. To explore this, we conducted FMTs from EAE mice to healthy controls (Ctr), and then evaluated barrier leakage and examined OCLN expression and subcellular localization at the blood-spinal cord barrier, BBB and blood-CSF barrier. Specifically, Ctr mice received consecutive daily FMTs from EAE mice (Ctr^EAE^) for 5 days, and these mice were then compared to Ctr recipients that received FMTs from control donor mice (Ctr^Ctr^) (Fig. 3A). To evaluate barrier leakage more sensitively, we utilized a 3 kDa BD tracer instead of the previously used 70 kDa BD tracer in Fig. 2, enabling the detection of even subtle changes in barrier integrity. In the spinal cord, a trend for increased leakage of BD was observed in Ctr^EAE^ compared to Ctr^Ctr^ (Fig. 3B, and D) and the EAE microbiome exerted deleterious effects on OCLN, as both the OCLN expression and the continuous OCLN length was significantly decreased in Ctr^EAE^ compared to Ctr^Ctr^ (Fig. 3C, E, and F). At the BBB, a trend for increased leakage of BD was also observed in healthy mice with EAE-derived FMT (Fig. 3G, and I), while OCLN expression remained unaltered in Ctr^EAE^ compared to Ctr^Ctr^ (Fig. 3H and J). However, the continuous OCLN length was still significantly decreased in Ctr^EAE^ *versus* Ctr^Ctr^ (Fig. 3H, and K). At the blood-CSF barrier, we still observed elevated (not significant trend) 3 kDa BD leakage (Fig. 3L, and N), but neither OCLN expression nor continuous length was affected in Ctr^EAE^ *versus* Ctr^Ctr^ (Fig. 3M, O-P). The evaluation of an additional TJ protein at the blood-CSF barrier, namely CDH1, did not show significant differences, although a trend towards decreased CDH1 expression was observed in healthy mice after receive gut microbiome from EAE mice (Figure S5). Taken together, these findings show that the EAE microbiome has a detrimental effect on OCLN-associated TJ expression and localization, which was most pronounced at the blood-spinal cord barrier. However, EAE microbiota did not significantly impact CNS barrier leakiness, although a trend for increased BD leakage was present at all three investigated CNS barriers.

### FMT of gut microbiota from healthy mice partially protects against EAE-induced loss of brain barrier integrity

**Fig. 4:**
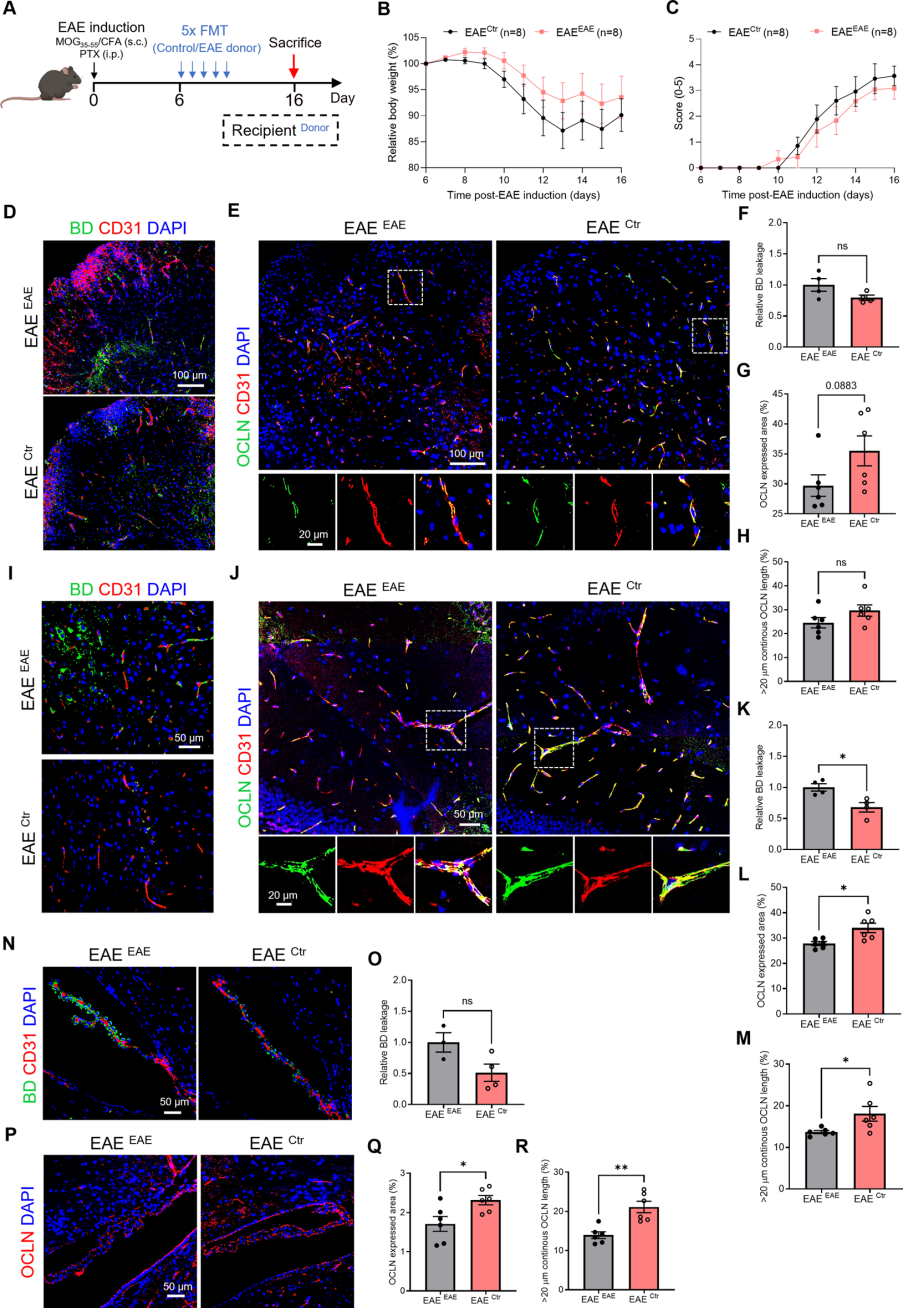
Gut microbiota from healthy mice exerts beneficial effects on CNS barrier integrity in EAE mice. (**A**) Schematic overview of the experimental set-up. Six days after EAE induction, mice were administered fecal contents from healthy control (Ctr) mice or EAE mice orally for 5 consecutive days, and mice were sampled on day 16. (B-C) Relative body weight (BW, %) (**B**) and clinical scores (**C**) of EAE mice after FMT with fecal content from Ctr or EAE mice (*n* = 8). (**D**-**E**) Representative images of immunohistochemical stainings for 3 kDa BD/CD31 (**D**) and OCLN/CD31 (**E**) on spinal cord sections. Scale bar 100 μm (**D**), 100 μm (up) and 20 μm (down) (**E**). (**F**-**H**) Quantification BD staining (**F**), OCLN expressed area (**G**) and OCLN continuous length of > 20 μm (**H**) in spinal cord tissue (*n* = 4–6). (**I**-**J**) Representative images for 3 kDa BD/CD31 (**I**) and OCLN/CD31 (**J**) stainings of BBB blood vessels in the cortex. Scale bar 50 μm (**I**), 50 μm (up) and 20 μm (down) (**J**). (**K**-**M**) Quantification BD staining (**K**), OCLN expressed area (**L**) and OCLN continuous length of > 20 μm (**M**) in BBB blood vessels in the cortex (*n* = 4–6). (**N**-**O**) Representative images for 3 kDa BD/ CD31 (**N**) and OCLN (**O**) stainings on ChP. Scale bar 50 μm. (**P**-**R**) Quantification BD staining (**P**), OCLN expressed area (**Q**) and OCLN continuous length of > 20 μm (**R**) in ChP (*n* = 3–6). Datapoints represent mean ± SEM. Statistical analysis was performed with unpaired t-test or Mann-Whitney test. * *p* < 0.05; ** *p* < 0.01; *** *p* < 0.001; **** *p* < 0.0001. BBB, blood-brain barrier; BD, biotinylated dextran; ChP, choroid plexus; CFA, complete Freund’s adjuvant; CNS, central nervous system; EAE, experimental autoimmune encephalomyelitis; FMT, fecal microbiota transplantation; Ctr, healthy control; MOG, myelin oligodendrocyte glycoprotein; OCLN, Occludin; PTX, pertussis toxin; TJs, tight junctions

To explore whether a healthy microbiome is able to prevent EAE-associated loss in brain barrier integrity, we administered the gut microbiome from control mice to EAE mice (EAE^Ctr^) for 5 consecutive days, starting from day 6 until day 10 p.i., followed by evaluation of CNS barrier leakage, OCLN expression and morphology analysis, six days after the last FMT (day 16 p.i.) (Fig. 4A). EAE mice that received FMT from EAE mice (EAE^EAE^), following the same treatment scheme as EAE^Ctr^, were included as controls (Fig. 4A). Fecal content from EAE donor mice was collected when a clinical score of at least 2 was reached. To verify that the microbiota composition was altered by the FMT procedure, we performed qPCR analysis comparing the microbiome before and after FMT (Figure S6). This confirmed partial engraftment, with a consistent shift in selected taxa in EAE recipient mice receiving healthy donor FMT, whereas no such shift was observed following EAE donor FMT. These findings support that the FMT influenced the host gut microbiome composition despite the absence of ABX pre-clearance.

As depicted in Fig. 4B-C, EAE mice that received FMT from healthy control mice (EAE^Ctr^) did not show a different weight loss curve or overall EAE disease course compared to EAE mice that received FMT from EAE mice (EAE^EAE^). In the spinal cord, there was no significant difference in 3 kDa BD leakage (Fig. 4D and F), along with consistent OCLN expression and continuous length (Fig. 4E and G-H) between EAE^Ctr^ and EAE^EAE^. Notably, it is worth mentioning that OCLN expression displayed a discernible increasing trend, albeit not achieving statistical significance (*p* = 0.0883). Remarkably, a significant barrier improvement was observed at the BBB, reflected by decreased BD leakage (Fig. 4I and K) and increased OCLN expression as well as continuous OCLN length in EAE mice upon FMT from Ctr mice compared to EAE^EAE^ mice (Fig. 4J and L-M). Similar effects were detected at the blood-CSF barrier, more specifically decreased trend in BD leakage but without significant change (Fig. 4N and P), and significantly increased OCLN expression and continuous length in EAE^Ctr^ compared to EAE^EAE^ (Fig. 4O, Q-R). In addition, CDH1 expression and > 20 μm continuous length distribution at the blood-CSF barrier were significantly increased in EAE^Ctr^ mice compared with EAE^EAE^ mice (Figure S7). However, despite the changes in barrier integrity, this was not correlated with changes in altered infiltration of CD3^+^ T-cells and IBA1^+^ cells in spinal cord and brain, respectively, between EAE^Ctr^ and EAE^EAE^ (Figure S8). Nevertheless, the level of CD3^+^ T-cell and IBA1^+^ cell infiltration in spinal cord, but not in brain, showed significantly negative correlation with OCLN expression (Figure S8). Altogether, these data show that the transfer of a healthy microbiome into mice can partially dampen EAE-induced barrier defects at TJ level at the BBB and blood-CSF barrier.

### Short-chain fatty acids (SCFAs) attenuate CNS barrier disruption in EAE mice

**Fig. 5:**
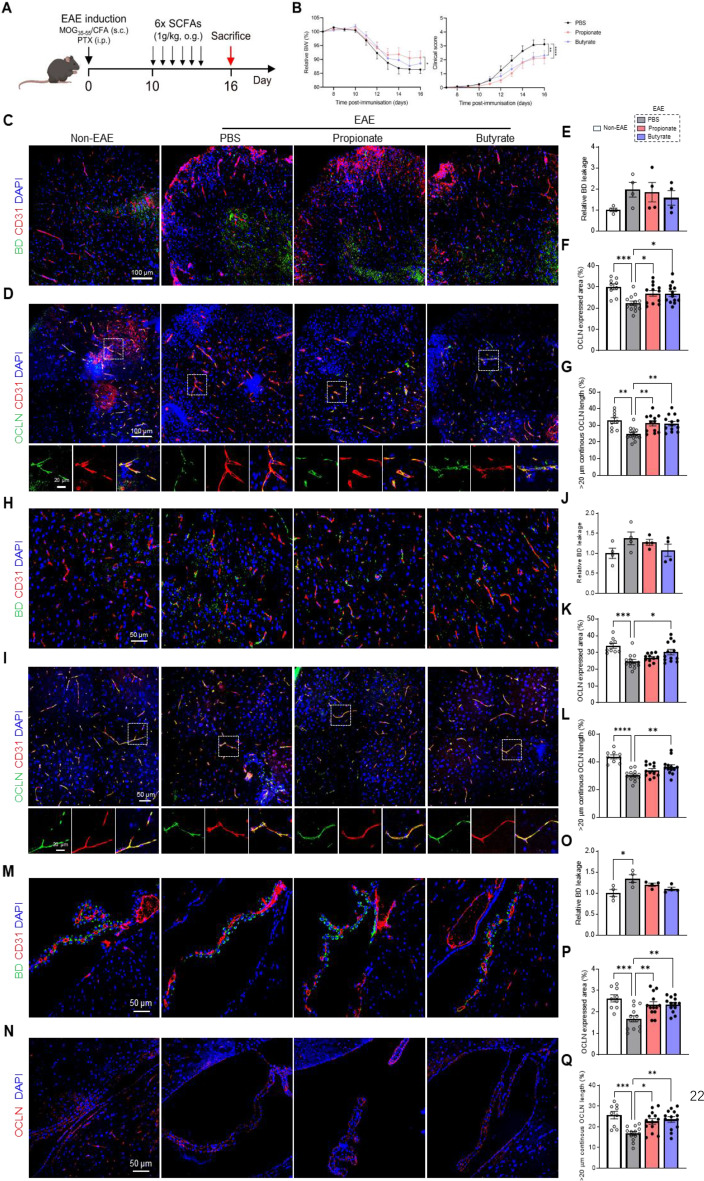
SCFA treatment reduce CNS barrier dysfunction in EAE mice. (**A**) Schematic overview of the experimental set-up. SCFA treatment (once per day for 6 days) was started 10 days after EAE induction. EAE mice were sampled at day 16 p.i. (**B**) Relative body weight (BW, %) (left) and clinical scores (right) of EAE mice treated with propionate, butyrate *versus* PBS (*n* = 15). (**C**-**D**) Representative images of immunohistochemical staining for 3 kDa BD/CD31 (**C**) and OCLN/CD31 (**D**) on spinal cord sections. Scale bar 100 μm (up) (**C**-**D**) and 20 μm (down) (**D**). (**E**-**G**) Quantification of BD staining (**E**), OCLN expressed area (**F**) and OCLN continuous length of > 20 μm (**G**) in spinal cord (*n* = 4–13). (**H**-**I**) Representative images for 3 kDa BD/CD31 staining (**H**) and OCLN/CD31 staining (**I**) of BBB blood vessels in the cortex. Scale bar 50 μm (up) (**H**-**I**) and 20 μm (down) (**I**). (**J**-**L**) Quantification of BD staining (**J**), OCLN expressed area (**K**) and OCLN continuous length of > 20 μm (**L**) in BBB blood vessels in the cortex (*n* = 4–13). (**M**-**N**) Representative images for BD/CD31 staining (**M**) and OCLN staining (**N**) in ChP tissue. Scale bar 50 μm. (**O**-**Q**) Quantification of BD staining (**O**), OCLN expressed area (**P**) and OCLN continuous length of > 20 μm (**Q**) in ChP (*n* = 4–13). Datapoints represent mean ± SEM. Statistical analysis was performed with two-way ANOVA (**B**) or one-way ANOVA (**E**-**G**, **J**-**L**, **O**-**Q**) followed by Tukey post hoc test). (*p < 0,05; ns: not significant). BBB, blood-brain barrier; BD, Biotinylated dextran; CFA, complete Freund’s adjuvant; ChP, choroid plexus; EAE, experimental autoimmune encephalomyelitis; MOG, myelin oligodendrocyte glycoprotein; SCFA, short-chain fatty acids; OCLN, occludin; o.g., oral gavage

SCFAs, the main metabolites produced by bacterial fermentation of dietary fiber in the gastrointestinal tract, are speculated to have a key role in microbiota-gut-brain crosstalk [38]. Propionate and butyrate are known to enhance the integrity of the BBB and blood-CSF barrier by facilitating TJ expression and assembly [14, 27, 39]. Hence, we studied whether propionate and butyrate might have the same beneficial effects in improving the integrity of CNS barriers in MS. In alignment with previous studies [40, 41], we validated a significant decrease in fecal propionic acid during EAE disease. Additionally, we observed a downward trend for butyrate levels is seen in the feces of EAE mice compared to those of healthy control mice (Figure S9). To evaluate whether SCFAs have restorative capacity on CNS barriers, we administered propionate or butyrate to EAE mice, starting from day 10 p.i. until day 15 p.i., followed by sampling at day 16 p.i. (Fig. 5A). As controls, EAE mice receiving PBS were included. Additionally, non-EAE mice administrated with PBS were taken along to evaluate the effect of SCFA treatment. The severity of EAE disease course was significantly attenuated in mice receiving propionate or butyrate, whereas only propionate supplementation significantly reduced body weight loss (Fig. 5B).

Non-EAE mice exhibited less BD leakage except in blood-spinal cord barrier, higher OCLN expression and more continuous OCLN length compared to EAE mice, indicating that EAE mice are associated with impaired CNS barrier integrity (Fig. 5C-Q). In the spinal cord, 3 kDa BD leakage was no changes between propionate- and PBS-treated EAE mice. However, it is noteworthy that butyrate-treated EAE mice exhibited a noticeable trend toward mitigated leakage (Fig. 5C and E). In addition, both propionate and butyrate treatment showed beneficial effect in improving OCLN expression and continuous length at the spinal cord (Fig. 5D and F-G). At the BBB, while both propionate- and butyrate-treated EAE mice did not show a reduction in BD leakage (Fig. 5H and J), a significant improvement of OCLN expression and continuous length was observed in butyrate-treated EAE mice (Fig. 5I and K-L). In case of the blood-CSF barrier, both propionate and butyrate treatment showed increased OCLN expression and improved OCLN continuous length distribution (Fig. 5N, P and Q), but both SCFAs did not exert effects on BD leakage at the ChP (Fig. 5M and O). Additionally, non-EAE mice exhibited higher CDH1 expression and better continuous CDH1 length at the blood-CSF barrier compared with EAE mice, but no barrier-restoring effect of SCFAs was observed in EAE mice (Figure S10). CD3^+^ T cell infiltration in the spinal cord and brain and IBA1^+^ cells in the spinal cord both trended down after SCFA treatment, suggesting that SCFA treatment was associated with changes in altered immune cell infiltration in the spinal cord and brain (Figure S11). Furthermore, the number of CD3^+^ T-cells and IBA1^+^ cells in both brain and spinal cord were significantly negative correlated with the level of OCLN expression at the corresponding regions (Figure S11). Notably, administration of SCFAs in healthy WT mice did not affect CD3^+^ T-cell infiltration or OCLN localization at the BBB or the blood-CSF barrier (Figure S12) and we did not observe a difference in the number of IBA1^+^ microglia in our previous study [35]. Altogether, these data suggest that SCFAs have the potential to at least partially prevent EAE-induced OCLN alterations at the different CNS barriers. To determine whether altered SCFA levels could explain the beneficial FMT effects observed in Fig. 3, we measured the propionate and butyrate levels in fecal samples from FMT recipient mice before the first FMT (pre-FMT) and 6 days after the last FMT (post-FMT). As displayed in Figure S13, this revealed that FMT didn’t result in significant differences in propionate and butyrate levels. This suggests that the barrier effects of FMT are not mediated by bulk changes in these SCFAs, although local or subtle metabolite shifts cannot be excluded.

## Discussion

Mounting evidence indicates that the gut microbiota influences the integrity and function of the central nervous system (CNS) barriers [13, 14, 27, 39], including the blood-brain (BBB), blood-cerebrospinal fluid (CSF) and blood-spinal cord barrier. These barriers meticulously control the passage of molecules and restrict the influx of inflammatory leukocytes from the periphery to the CNS, crucially maintaining CNS homeostasis [8, 42]. Notably, CNS barrier dysfunction holds a pivotal role in the pathology of numerous neurological diseases, such as Alzheimer’s disease (AD), Parkinson’s disease (PD) and multiple sclerosis (MS) [43–45]. In the context of MS, compromised CNS barrier integrity can facilitate leukocyte infiltration, culminating in the emerge of inflammatory demyelinating lesions [46]. Recent studies point to an important role of the gut microbiome and gut–brain axis in shaping both the permeability and maturation of CNS barriers under homeostasis, even though the exact mechanisms involved remain to be clarified.

Our investigation revealed that when mice were subjected to EAE, a well-established murine model for MS, and their gut microbiota was depleted through ABX treatment, the resulting illness displayed reduced severity, with mitigated body weight loss, delayed symptom onset, and lower cumulative scores. Importantly, ABX treatment in healthy mice did not alter the composition of circulating immune cells, suggesting that the protective effects observed during EAE are not simply due to broad changes in baseline immune profiles. However, while this supports the idea that ABX treatment may not broadly alter immune profiles under homeostatic conditions, it does not exclude the possibility that ABX impact immune cell priming or activation specifically during EAE. Indeed, some of the ABX used in our cocktail, such as ciprofloxacin [47], have been shown to exert direct immunomodulatory effects, including modulation of T-cell function, independent of their microbiota-depleting properties. Therefore, while our data are consistent with a microbiota-mediated effect on EAE progression, we cannot definitively rule out additional microbiota-independent mechanisms, including direct effects of ABX on immune or glial cells. More broadly, it is important to acknowledge that the impact of ABX in EAE is not uniform: different classes vary in their ability to alter microbial composition and bacterial load, and timing of exposure is critical. Whereas treatment in adult mice can attenuate disease severity, early-life ABX exposure has been associated with worsened EAE [24], underscoring the importance of microbial resilience and developmental context. Beyond immune modulation, microbiota perturbation may also influence CNS physiology through non-immune mechanisms, including vagal and sympathetic signaling pathways and the action of microbial-derived metabolites. Taken together, these considerations suggest that the protective effects we observed following broad-spectrum ABX treatment likely reflect a complex interplay of microbiota-dependent and microbiota-independent processes, which warrants further mechanistic dissection in future studies.

Our findings align with existing research reporting milder EAE disease presentation following microbiome depletion through ABX treatment and concurs with the observation that germ-free (GF) mice exhibit resistance to spontaneous EAE development in transgenic SJL/J mice [48–50]. Further mechanistic studies, such as antibiotic treatment in germ-free EAE mice or the use of individual ABX, would be needed to disentangle microbiota-dependent from microbiota-independent effects. Importantly, our study demonstrates that the depletion of microbiota through ABX treatment can beneficially impact the loss of CNS barrier integrity associated with EAE. This was characterized by the rearrangement of OCLN tight junction (TJ), evident in alterations in the covered area of the TJ protein occludin (OCLN). Previous work, including studies from our lab, has shown that the gut microbiota is essential for the formation of tight CNS barriers. In the absence of microbiota, through germ-free housing and via ABX treatment, both the BBB and blood–CSF barrier showed reduced expression of tight junction proteins, including occludin and claudins, which was reverted upon gut recolonization [11, 14, 51]. At first sight, these findings seem at odds with our observations in EAE, where ABX treatment is linked to attenuated barrier disruption. However, this apparent discrepancy highlights the context-dependent nature of microbiota–barrier interactions: while a diverse microbial community is essential for barrier maturation and maintenance under healthy conditions, disease-associated alterations in microbial composition during autoimmune inflammation may themselves promote barrier dysfunction, making their depletion protective.

EAE mice exhibit significant microbiota alterations early in the course of disease onset compared to healthy controls [20, 52, 53]. For instance, a decrease in the prevalence of commensal bacteria from the *Lactobacillaceae* family and an expansion of bacteria within the Clostridia class was observed in EAE mice [20, 53]. Similarly, among MS patients, gut dysbiosis is evident, characterized by altered levels of specific bacterial taxa [17, 54]. Despite variations in findings across microbiota assessment studies in MS patients, the consensus is that MS-associated microbiota promotes pro-inflammatory responses, including a skew towards pro-inflammatory T-cells differentiation over regulatory T-cells [55]. Therefore, we posit that the advantageous impact of ABX-induced microbiota depletion on barrier integrity within the EAE model may arise from its ability to prevent shifts in microbiota composition linked with EAE disease progression, which in turn influences a more favorable immune cell composition. Alterations in gut microbiota composition have been linked to MS risk, course and progression, and fecal microbiota transplant (FMT) with healthy gut microbiota has been shown to modulate inflammatory responses and alleviate EAE progression [56, 57]. In our study, we conducted FMTs between EAE mice and healthy recipient, assessing CNS barrier integrity. These FMT procedures caused a consistent change in the microbiota composition of EAE recipient mice who received healthy control donor FMT, but as expected not in those who received EAE donor FMT. It should be noted that we did not perform ABX clearance of the gut before FMT, which is often used to increase engraftment. We deliberately avoided this, as broad-spectrum ABX treatment is known to disrupt CNS barrier integrity, which would have confounded our analyses. Importantly, this type of ABX pre-treatment is not directly comparable to the prolonged broad-spectrum ABX regimens used in our study. While qPCR confirmed engraftment of selected taxa, residual recipient microbiota and cage-level variation may have influenced outcomes. Our findings should therefore be interpreted as evidence of directional microbiota effects rather than complete community transfer. As an alternative approach, polyethylene glycol (PEG) pre-treatment could be considered in future work. PEG has been shown to facilitate FMT engraftment by lowering bacterial load, although it does not affect mucosa-associated bacteria. In addition, future studies in germ-free hosts could be of interest to further substantiate our results.

Despite the absence of detectable differences in overall disease severity in this set-up, we observed effects on CNS barrier integrity. We found that compromised TJs in the CNS barriers of EAE mice could be partially restored by introducing gut microbiota from healthy donors through transplantation. Conversely, introducing fecal microbiota from EAE mice to healthy mice resulted in a decline in CNS barrier integrity. These findings align with prior research from our group and others demonstrating that impaired CNS barrier characteristics observed in ABX-treated and GF mice could be normalized by reintroducing normal gut microbiota [11, 14]. Furthermore, our results underscore that distinct gut microbiota compositions may have opposing impacts, as the EAE-associated microbiome negatively affects a healthy CNS barrier, while normal microbiota beneficially influences compromised barriers in EAE. It is noteworthy that we did not assess the influence of FMT on gut barrier permeability. Both MS patients and EAE mice have exhibited impaired intestinal integrity [58, 59], potentially leading to a leaky gut and bacterial translocation to the systemic circulation, which could explain the increased levels of lipopolysaccharides (LPS) observed in the plasma of MS patients or EAE mice [60, 61]. Such low-grade systemic inflammation can, in turn, adversely affect CNS barrier integrity. Given that increased intestinal permeability can stem from gut dysbiosis [62], an FMT of EAE-microbiota to healthy mice could cause gut barrier deficits that subsequently impact CNS barrier integrity. We acknowledge that our study did not examine whether FMT influenced gut-associated immune cell activation or mucosal immune responses, which may in turn impact CNS barrier function. Future studies using FITC-dextran assays or plasma LPS quantification will be required to directly address this possibility. Moreover, flow cytometric analyses of immune cell subsets in the gut, spleen, and CNS at the peak of disease could help clarify how FMT shapes systemic and local immune responses relevant to barrier regulation. Additionally, we did not evaluate intestinal permeability or systemic LPS levels following FMT. It remains plausible that FMT modifies the gut microbiota in a way that reduces gut leakiness and systemic LPS translocation, thereby indirectly preserving CNS barrier integrity.

In addition to the diminished disruption of the CNS barriers, we observed that ABX-mediated microbiome depletion is associated with a reduced amount of CD3^+^ T cells and IBA1^+^ macrophages/microglia in the brains and spinal cords of EAE mice. Correspondingly, previous reports noted that ABX-treated EAE mice exhibited decreased populations of pro-inflammatory T-cells and macrophages, along with increased regulatory T-cell counts in the peripheral organs [49, 50]. However, we did not observe changes in the number of infiltrated T-cells or macrophages in EAE mice that received fecal microbiota from healthy donors. It is worth noting that the question whether barrier dysfunction precedes or results from leukocyte infiltration into the CNS in MS research remains under debate. On the one hand, barrier permeability can exist independently from immune cell infiltration; for instance, the enhancement of BBB tight junctions through claudin-1 has been shown to ameliorate chronic EAE disease and reduce BBB permeability without affecting immune cell trafficking into the CNS [63]. On the other hand, infiltrating leukocytes release inflammatory mediators that can trigger barrier disruption, promoting further leukocyte influx [64, 65]. In our study, the observation that microbiota transfer affects CNS barrier properties to some extent without necessarily impacting immune cell infiltration, suggests that these two aspects might not be simultaneous occurrences. However, our data are associative and do not allow causal inference regarding whether barrier dysfunction precedes or results from immune infiltration. Temporal analyses at earlier time points will be required to address this question. The notion that immune activation and barrier integrity could operate independently gains additional support through our Pearson correlation analysis of OCLN expression and the percentage of CD3^+^ T cells/IBA1^+^ microglia at the spinal cord and brain borders. Across various experimental conditions a negative correlation emerges between these two factors.

The gut microbiota composition in MS patients is characterized by a decline in abundance of bacteria, such as *Clostridia*, that generate short-chain fatty acid (SCFA) [30, 66, 67]. This aligns with findings of reduced SCFA levels in the blood and feces of MS patients [66, 68]. Recent studies have shown that metabolites produced by gut microbiota, particularly SCFAs, play crucial signaling roles that can influence the integrity of the BBB and blood-CSF barrier, and overall brain function [27, 39]. In line with earlier studies [40, 41], we confirmed a notable reduction in the levels of SCFA-producing bacteria, as indicated by the relative expression ratio of *Firmicutes* to *Bacteroides*, and a decrease in fecal propionic acid levels during EAE disease. Although SCFAs are recognized as one of the pathways through which the microbiota can influence CNS barriers [35], they might not represent the primary mechanism for the FMT-induced enhancement of CNS barrier integrity, as evidenced by unchanged propionate and butyrate levels following FMT. However, the SCFA changes may be too subtle for detection in fecal samples. Therefore, while local SCFA alterations cannot be ruled out, there is insufficient evidence to support significant differences in SCFA levels caused by FMT and accountable for the FMT-induced effects on the brain. Yet, orally treated EAE mice with sodium propionate or sodium butyrate at a dosage of 10.4 mM and 9.31 mM/kg body weight respectively, starting at day 10 p.i. until day 15 p.i., resulted in a milder EAE clinical score upon short-term propionate and butyrate treatment, which is in agreement with previously documented beneficial effects of long-term SCFA treatment-initiated weeks before EAE induction. Furthermore, it is important to note that preceding studies applied different SCFA doses compared to our study, i.e. a mixture of 80 mM acetate, 40 mM propionate, and 20 mM butyrate or 200 mM of these individual SCFAs in the drinking water [68, 69]. In our study, propionate and butyrate treatment protected against the EAE-associated disturbed TJ localization at all CNS barriers. Similar beneficial effects of propionate and butyrate on BBB and blood-CSF barrier integrity were reported in an in vitro study using hCMEC/D3 cell cultures and an in vivo study using mice lacking gut microbiota [14]. Mechanistically, the FFAR3 receptor on the BBB has been identified as a pivotal pathway for gut-brain interactions [11, 27]. Alongside this direct interaction, the vagus nerve plays a crucial role in gut-brain communication by indirectly transmitting signals from the gut microbiota and SCFAs to the brain, as we and others previously reported [14, 70]. Furthermore, our findings show that butyrate treatment exhibits a more potent ability to enhance the expression and localization of TJ protein in the BBB compared to propionate. This observation aligns with our previous study in an AD mouse model [14]. At the other investigated CNS barriers, effects of both propionate and butyrate on OCLN expression and localization were similar. These differences may reflect variations in SCFA receptor expression (e.g., FFAR3 at the BBB but not detected in choroid plexus), differential metabolite stability in circulation (butyrate being more stable than propionate), or differences in uptake efficiency by barrier cells. Further work comparing receptor distribution and transport kinetics will be needed to clarify these mechanistic distinctions.

Indeed, our previous study showed that butyrate is more stable in the blood than propionate. Additionally, butyrate was found to cross the blood-CSF barrier in vitro, although neither butyrate nor propionate were detected in the mouse CSF, possibly due to low concentrations that current methods cannot detect [14]. Of note, while the blood-spinal cord barrier structurally resembles the BBB [8], it does not exhibit the same barrier-enhancing response to butyrate treatment. Consequently, further investigations comparing the disparities in SCFA receptors in the BBB and blood-spinal cord barrier are necessary. Additionally, understanding how SCFAs act on blood-CSF barrier requires further exploration, as we did not detect the SCFA receptors on ChP of mice in our previous study [14]. Important to note is that our study did not investigate changes in other microbiota-derived metabolites besides SCFAs. Recent studies have shown that vancomycin, a component of our ABX cocktail, alters tryptophan/indole and bile acid metabolism, which may also contribute to EAE outcomes and CNS barrier function [71]. These pathways warrant further targeted metabolomic analysis in future studies.

Moreover, alongside SCFA-dependent mechanisms, SCFA-independent effects may also contribute to the outcomes observed after FMT. These could involve altered gut barrier permeability leading to systemic endotoxemia, as lipopolysaccharides have been linked to CNS barrier dysfunction in both MS patients and EAE models. In addition, gut microbes modulate bile acid metabolism and tryptophan metabolism, which may influence endothelial and epithelial junctions. Microbiota-driven modulation of mucosal or systemic immune responses, as well as signaling through vagal and sympathetic neural pathways, could further impact barrier regulation. Future studies employing targeted metabolomics, gut permeability assays (e.g., FITC-dextran), and plasma LPS quantification will be essential to dissect these mechanisms.

It is crucial to underscore that the outcomes of microbiome manipulations across our various experimental setups did not consistently result in uniform effects on the distinct CNS barriers. While the depletion of microbiome through ABX treatment notably benefited the BBB and blood-spinal cord barrier, the introduction of healthy microbiota to EAE mice predominantly exhibited a positive influence on the blood-CSF barrier and BBB integrity. Furthermore, the adverse effects on CNS barriers resulting from the transfer of fecal microbiota from EAE mice to healthy counterparts, were predominantly evident at the spinal cord. This divergence might stem from variations in the timing of microbiota manipulation; ABX served as a prophylactic treatment initiated before EAE induction, whereas FMTs were performed during the EAE pre-onset stage. Additionally, the distinct CNS barriers may exhibit varying degrees of sensitivity to microbiota-related effects.

At the same time, several limitations of our work—including the possibility of microbiota-independent ABX effects, partial engraftment following FMT, and small sample sizes in some experiments—underscore the need for cautious interpretation. While our study is preclinical and requires further validation, it highlights the potential of microbiota-targeted approaches as promising avenues to strengthen CNS barriers in MS. Future translational and clinical research will be essential to define how such strategies — whether through microbial metabolites, dietary modulation, or tailored microbiota interventions — might be safely and effectively harnessed to complement existing therapies.

Altogether, our findings suggest that the gut microbiota influences CNS barrier properties in EAE, with SCFAs capable of partially restoring tight junction organization. These observations align with emerging literature identifying gut-derived signals as modulators of neuroimmune homeostasis. While our study is preclinical and requires further validation, it highlights the potential of microbiota-targeted approaches as promising avenues to strengthen CNS barriers in MS. Future translational and clinical research will be essential to define how such strategies — whether through microbial metabolites, dietary modulation, or tailored microbiota interventions — might be safely and effectively harnessed to complement existing therapies.

## Electronic supplementary material

Below is the link to the electronic supplementary material.

Supplementary Material 1

## Data Availability

Data is provided within the manuscript or supplementary information files.

## Abbreviations

AD: Alzheimer’s disease
ABX: Antibiotics
BBB: Blood-brain barrier
BCSF: Blood-cerebrospinal fluid
CNS: Central nervous system
ChP: Choroid plexus
CDH1: E-cadeherin
EAE: Experimental autoimmune Encephalomyelitis
FMT: Fecal microbiome transplant
FMO: Fluorescence-minus-one
FFAR: Free fatty acid receptor
GF: Germ free
Ctr: Healthy controls
LPS: Lipopolysaccharides
MS: Multiple sclerosis
ns: Not significant
OCLN: Occludin
PD: Parkinson’s disease
PTX: Pertussis toxin
p.i.: Post-immunization
RR: Relapsing-remitting
SCFA: Short-chain fatty acids
SPF: Specific pathogen-free
SpC: Spinal cord
TJ: Tight junction
