# Supplementary material for: Gut microbiota modulates CNS barrier function in murine model for multiple sclerosis
Source: Fluids Barriers CNS. 2025 Nov 7;22:114. doi: 10.1186/s12987-025-00724-y (PMC12595800; doi:10.1186/s12987-025-00724-y)
Supplement: Supplementary file 1 — Supplementary Material 1 [file 12987_2025_724_MOESM1_ESM.docx]

**SUPPLEMENTARY FIGURES**

**
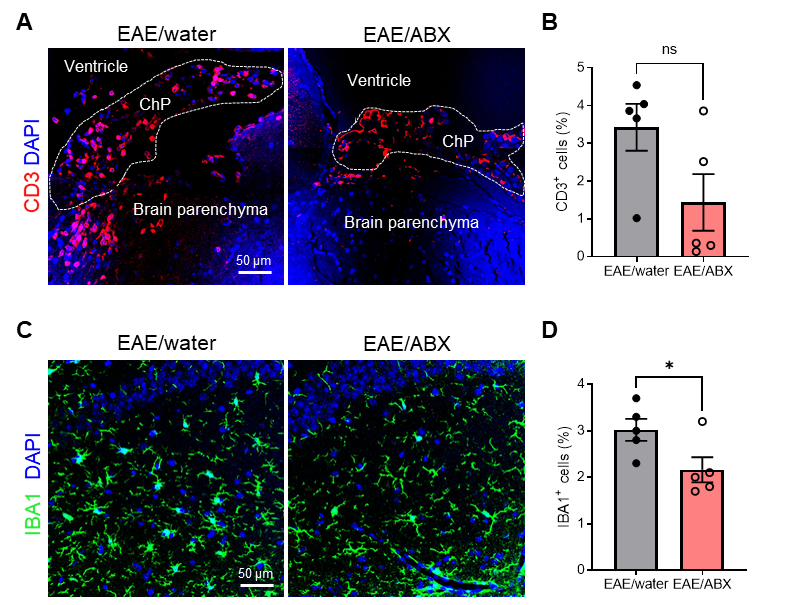
**

**Supplementary Figure S1. CD3^+^ and IBA1^+^ cells in the brain of ABX treated and control treated EAE mice**. **(A)** Representative images of CD3 staining on brain. Scale bar 50 µm. **(B)** Quantification CD3^+^ cells in brain tissue (n = 5). **(C)** Representative images of IBA1 staining on brain. Scale bar 50 µm. **(D)** Quantification IBA1^+^ cells brain tissue (n = 5). Datapoints represent mean ± SEM. Statistical analysis was performed with unpaired t-test (**p* < 0.05; ns: not significant). ABX, antibiotics; EAE, experimental autoimmune encephalomyelitis.


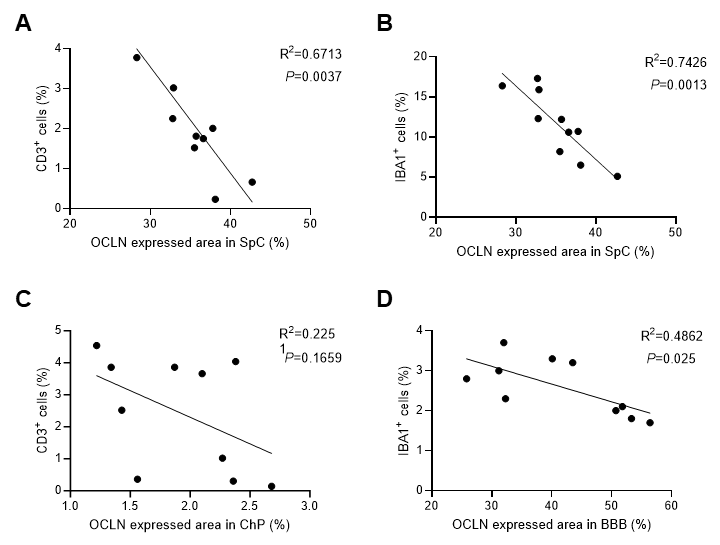


**Supplementary Figure S2. Pearson correlation analysis of TJ expression in CNS barriers and immune cell infiltration**. **(A-B)** The relationship between OCLN expression and % CD3^+^ cells of all nuclei **(A)** and IBA1^+^ cells **(B)** in spinal cord. **(C)** The relationship between OCLN expression and % CD3^+^ cells of all nuclei in blood-CSF barrier. **(D)** The relationship between OCLN expression and IBA1^+^ cells in BBB. OCLN, occludin; SpC, spinal cord; TJ, tight junction.


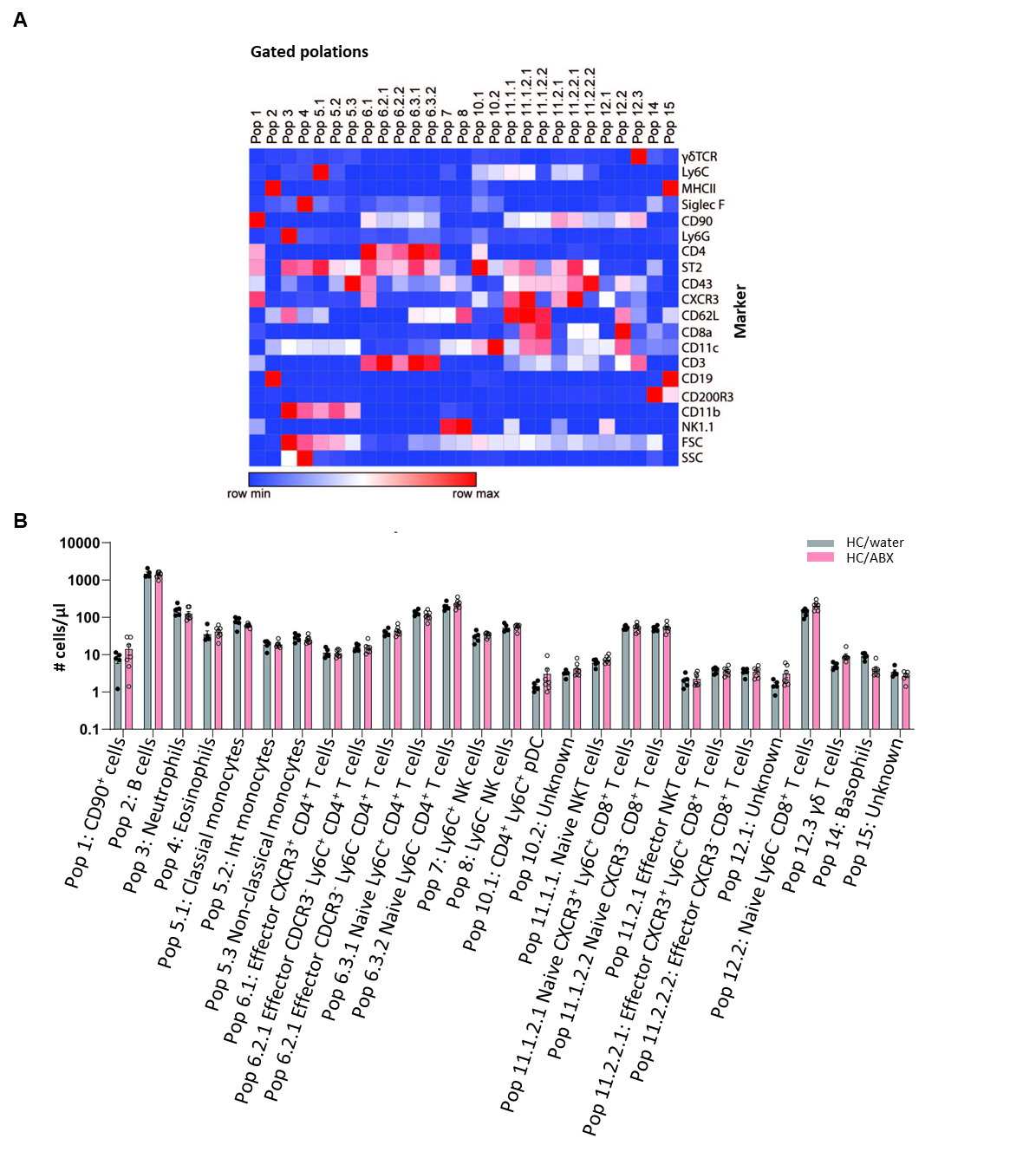


**Supplementary Figure S3. Immunophenotyping of the blood from ABX treated and control treated mice.** Unsupervised flow cytometry analysis of single CD45^+^ immune cells derived from blood samples of mice receiving antibiotics (ABX) in their drinking water for two weeks compared to those that receiving regular water. (**A**) Identification of different immune cell population based on marker expression. (**B**) Absolute number of cells/µl blood for each identified immune cell population. Data presented as mean ± SEM (n = 7/group). Statistical analysis by two-way ANOVA with Tukey post hoc multiple comparison.


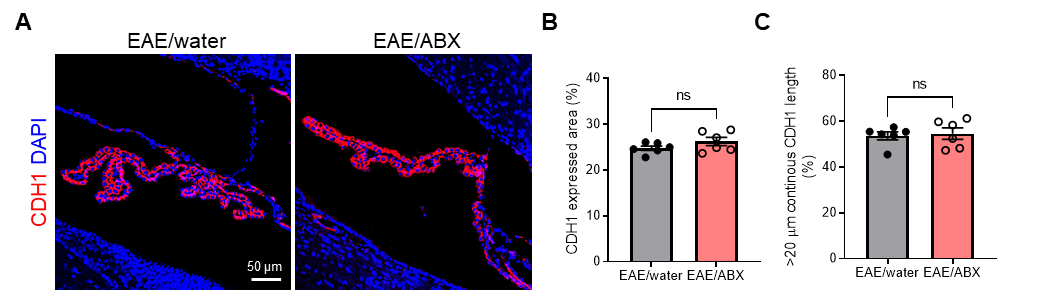
**Supplementary Figure S4. The impact of gut microbiota on blood-CSF barrier in EAE mice**. **(A)** Representative images of CDH1 staining on ChP. Scale bar 50 µm. **(B-C)** Quantification CDH1 expressed area (B) and CDH1 continuous length of > 20 µm (C) in ChP (n = 6). Datapoints represent mean ± SEM. Statistical analysis was performed with unpaired t-test (ns: not significant). ABX, antibiotics; EAE, experimental autoimmune encephalomyelitis; OCLN, Occludin; CDH1, E-cadherin.


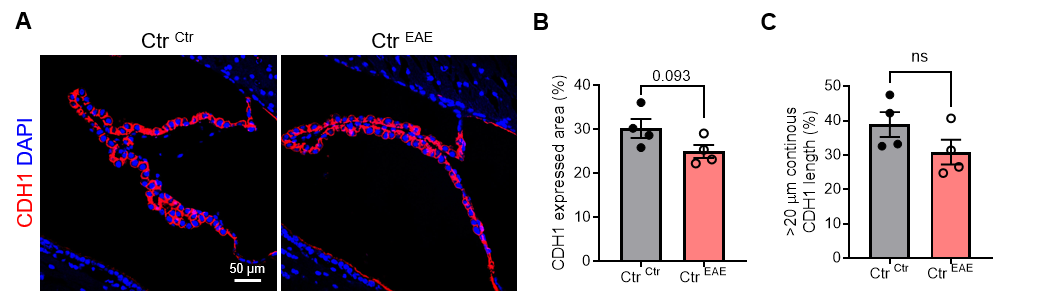


**Supplementary Figure S5. The impact of FMT on blood-CSF barrier in healthy mice**. **(A)** Representative images of CDH1 staining on ChP. Scale bar 50 µm. **(B-C)** Quantification CDH1 expressed area (B) and CDH1 continuous length of > 20 µm (C) in ChP (n = 4). Datapoints represent mean ± SEM. Statistical analysis was performed with unpaired t-test (ns: not significant). CDH1, E-cadherin; ChP, choroid plexus; CSF, cerebrospinal fluid; FMT, fecal microbiome transplant.

**Supplementary Figure S6. The influence of FMT on microbiome composition of the recipient mice.** Microbiome composition before (grey dots) and after FMT (pink dots) in EAE recipients which received fecal material from EAE donor mice or Ctr donor mice. EAE, experimental autoimmune encephalomyelitis; FMT, fecal microbiota transfer; Ctr, healthy control.


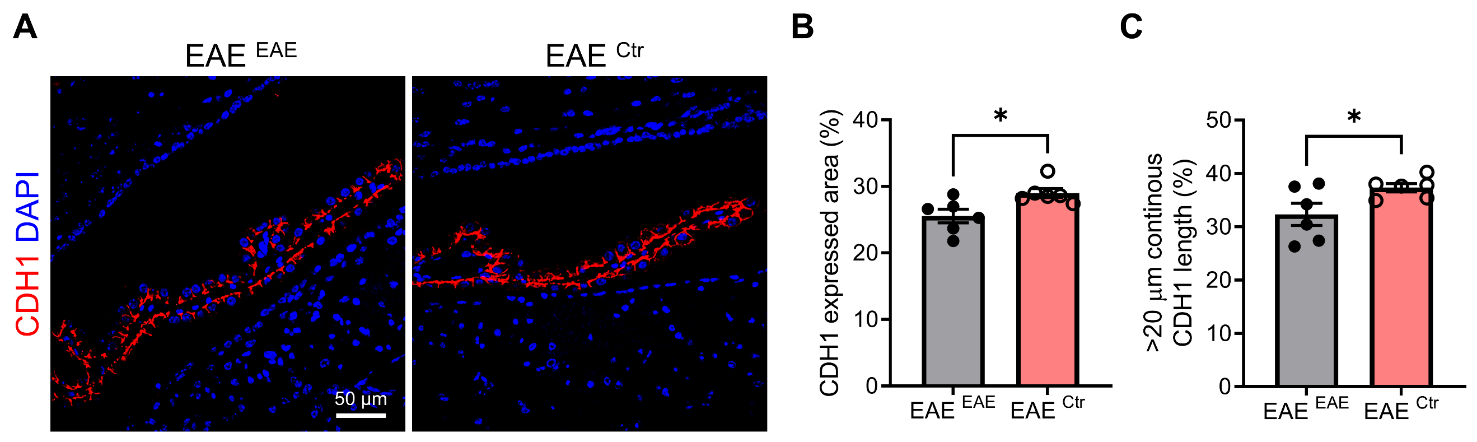
**Supplementary Figure S7. The impact of FMT on blood-CSF barrier in EAE mice**. **(A)** Representative images of CDH1 staining on ChP. Scale bar 50 µm. **(B-C)** Quantification CDH1 expressed area (B) and CDH1 continuous length of > 20 µm (C) in ChP (n = 6). Datapoints represent mean ± SEM. Statistical analysis was performed with unpaired t-test. CDH1, E-cadherin; ChP, choroid plexus; CSF, cerebrospinal fluid; EAE, experimental autoimmune encephalomyelitis; FMT, fecal microbiome transplant.


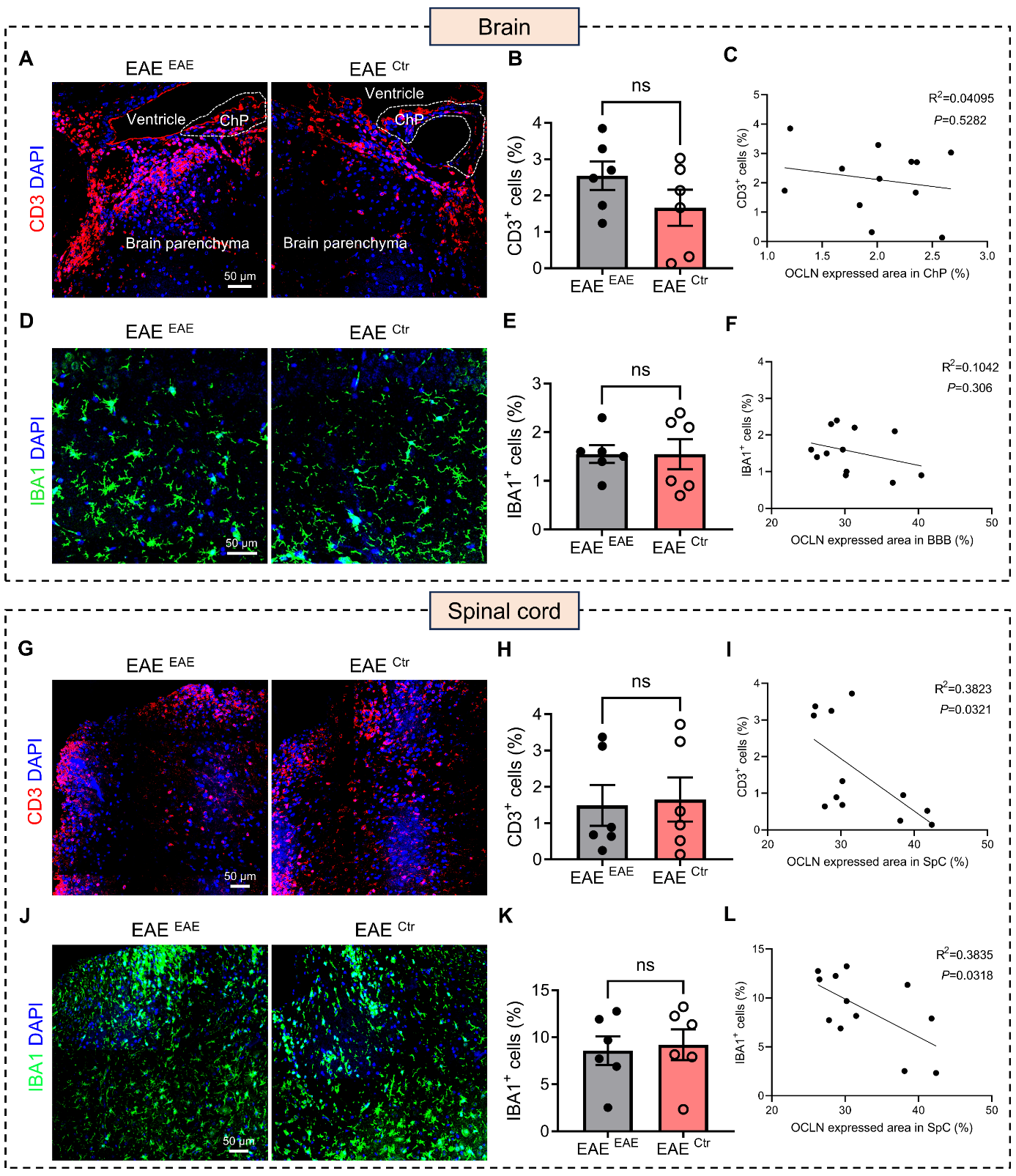


**Supplementary Figure S8. The impact of gut microbiota on the populations of CD3^+^ and IBA1^+^ cells in the brain and spinal cord of EAE mice receiving a fecal microbiota transplant (FMT) from EAE or Ctr mice**. **(A-B)** Representative images (A) of CD3 staining (red) on brain sections and the corresponding quantification (B) (n = 6). Scale bar represents 50 µm. **(C)** Pearson correlation analysis of OCLN expression and CD3^+^ cells at the blood-CSF barrier. **(D-E)** Representative images of IBA1 (green) on brain sections (D) and the corresponding quantification (E) (n = 6). Scale bar represents 50 µm. **(F)** Pearson correlation analysis of OCLN expression and IBA1^+^ cells at the BBB. **(G-H)** Representative images (G) of CD3 staining (red) and corresponding quantification of the amount of CD3^+^ cells (H) on spinal cord sections (n = 6). Scale bar represents 50 µm. **(I)** Pearson correlation analysis of OCLN expression and CD3^+^ cells in spinal cord. **(J-K)** Representative images (J) of IBA1 staining (green) and corresponding quantification of IBA1 + cells on spinal cord sections (n = 6). Scale bar 50 µm. **(L)** Pearson correlation analysis of OCLN expression and IBA1^+^ cells in spinal cord. Datapoints represent mean ± SEM. Statistical analysis was performed with unpaired t-test (ns: not significant). BBB, blood-brain barrier; ChP, choroid plexus; EAE, experimental autoimmune encephalomyelitis; Ctr, healthy controls; OCLN, occluding; SpC, spinal cord.


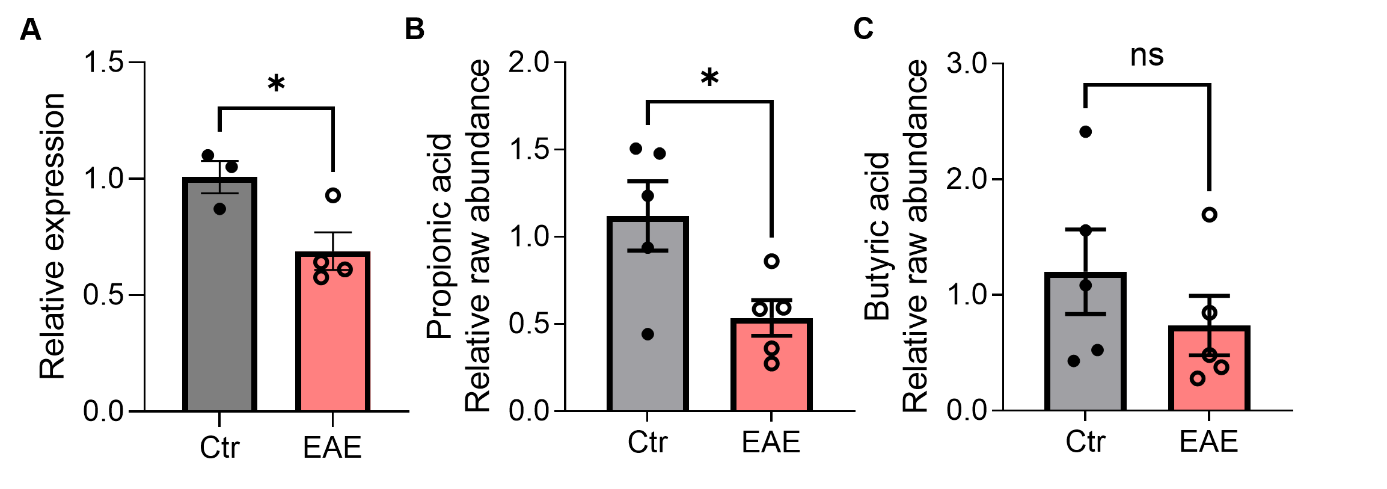


**Supplementary Figure S9. The levels SCFA producing bacteria and SCFA in fecal pellets of EAE mice.** (**A**) qPCR analysis of the gut microbiota of healthy control (Ctr) and experimental autoimmune encephalomyelitis mice (EAE) reveal a decrease in relative Firmicutes to Bacteroides ratio in EAE mice compared to Ctr. (**B-C**) Fecal propionic acid (B) and butyric acid (C). Datapoints represent mean ± SEM. Statistical analysis was performed with unpaired t-test (n = 5; one section per mouse). (**p* < 0,05; ns: not significant). EAE, experimental autoimmune encephalomyelitis; Ctr, healthy control; SCFA, short-chain fatty acids.


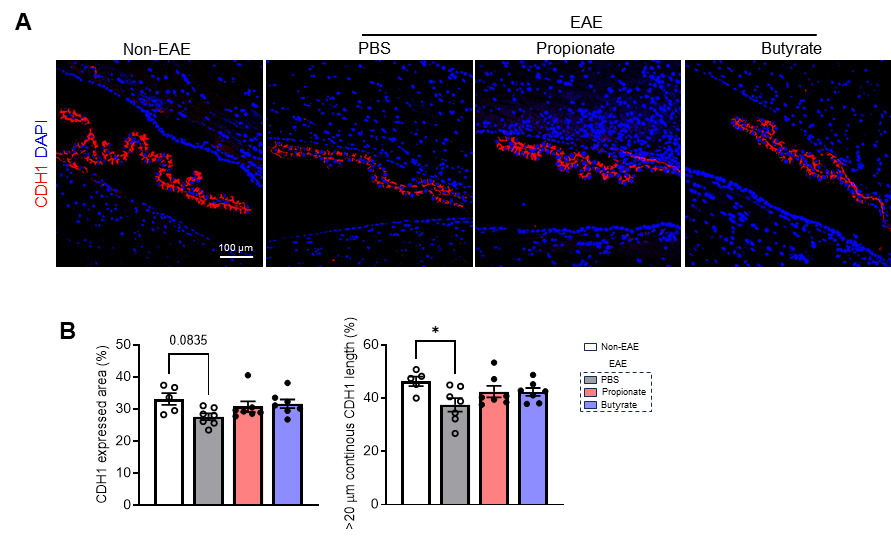


**Supplementary Figure S10. The impact of SCFA on blood-CSF barrier in EAE mice**. **(A)** Representative images of CDH1 staining on ChP. Scale bar 50 µm. **(B)** Quantification CDH1 expressed area (left) and CDH1 continuous length of > 20 µm (right) in ChP (n = 5–7). Datapoints represent mean ± SEM. Statistical analysis was performed with one-way ANOVA followed by Tukey post hoc test. CDH1, E-cadherin; ChP, choroid plexus; CSF, cerebrospinal fluid, EAE, experimental autoimmune encephalomyelitis; SCFA, short-chain fatty acid.


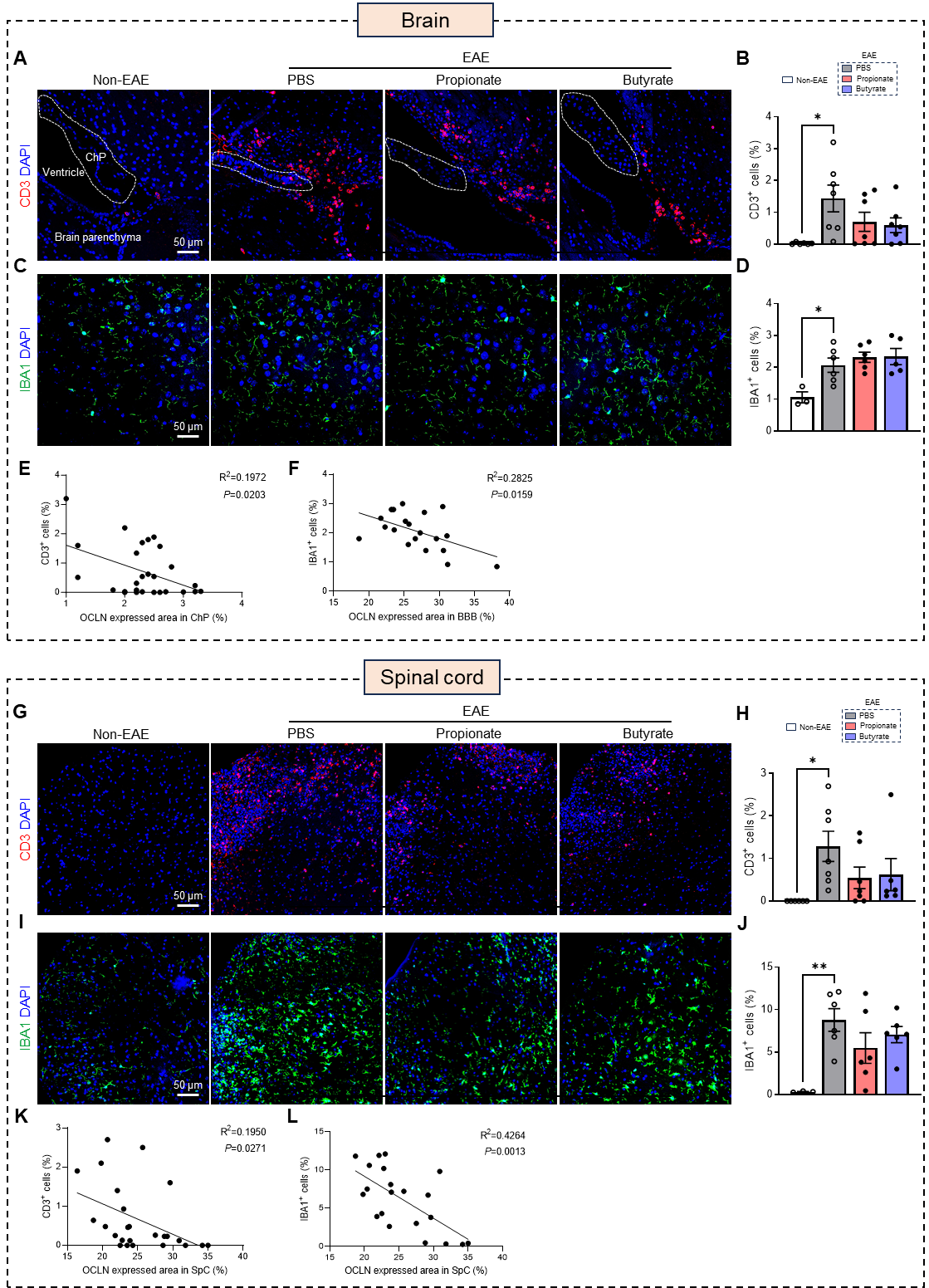


**Supplementary Figure S11. The impact of the SCFAs propionate and butyrate on the populations of CD3^+^ and IBA1^+^ cells in the brain and spinal cord of EAE mice**. **(A-B)** Representative images of CD3 (red) staining (A) and quantification (B) of the CD3^+^ cells in brain sections (n = 6–7). Scale bar represents 50 µm. **(C-D)** Representative images of IBA1 (green) staining (C) and quantification (D) of the amount IBA1^+^ cells in brain sections (n = 3–6). Scale bar represents 50 µm. **(E)** Pearson correlation analysis of OCLN expression and CD3^+^ cells at the blood-CSF barrier. **(F)** Pearson correlation analysis of OCLN expression and IBA1^+^ cells at the BBB. **(G-H)** Representative images (G) of CD3 (red) staining and quantification (H) CD3^+^ cells in spinal cord tissue (n = 6–7) on spinal cord. Scale bar represents 50 µm. **(I-J)** Representative images (I) of IBA1 (green) staining and quantification (J) of the amount of IBA1^+^ cells in spinal cord tissue (n = 6–7). Scale bar represents 50 µm. **(K-L)** Pearson correlation analysis of OCLN expression and CD3^+^ cells (K) and IBA1^+^ cells (L) in spinal cord. Datapoints represent mean ± SEM. Statistical analysis was performed with unpaired t-test. EAE, experimental autoimmune encephalomyelitis; Ctr, healthy controls; OCLN, occludin; SpC, spinal cord; SCFA, short-chain fatty acid.


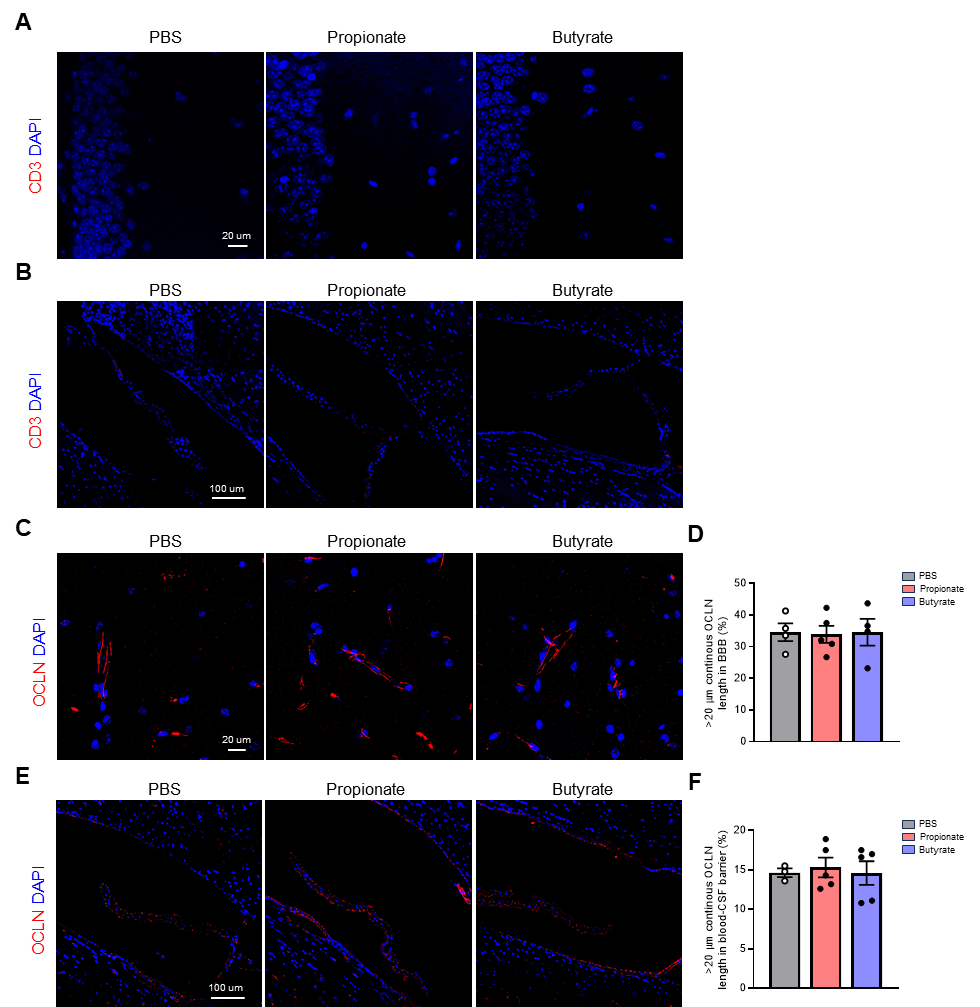


**Supplementary Figure S12. The impact of SCFAs on the amount of CD3^+^ cells and the tight junction (TJ)- associated OCLN at the brain barriers in healthy wild type (WT) mice**. **(A, B, C, E)** Representative images of CD3 (A, B; n = 4–5) and OCLN (C, E; n = 3–6) staining at the hippocampus (A, C; scale bar 20 µm) and the lateral ventricle blood-CSF barrier (B, E; scale bar 100 µm) of mice that were supplemented via oral gavage for three consecutive days with PBS, sodium propionate (1 g/kg body weight) or sodium butyrate (1 g/kg body weight). **(D, F)** The percentage of OCLN continuous length of > 20 µm in the hippocampus (D) and the blood-CSF barrier (F). Datapoints represent mean ± SEM. Statistical analysis was performed with unpaired t-test. CSF, cerebrospinal fluid; OCLN, Occludin; SCFA, short-chain fatty acid.


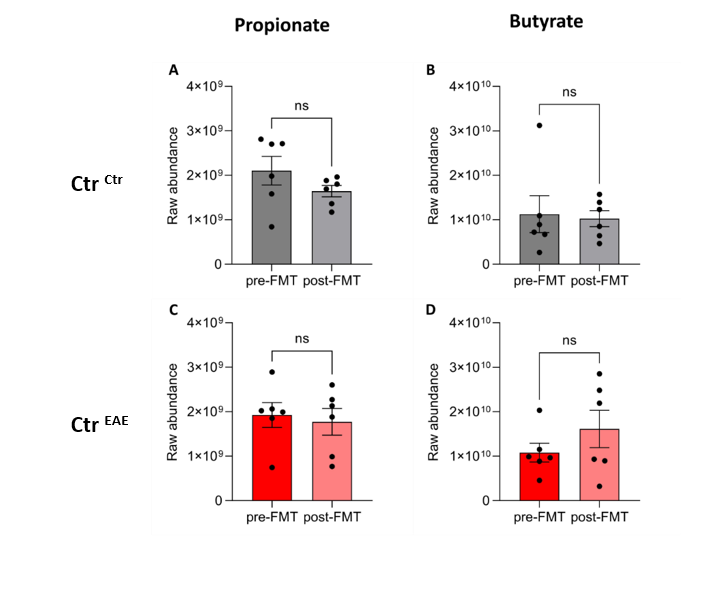


**Supplementary Figure S13. Fecal propionate and butyrate levels are not altered upon fecal microbiota transfer (FMT).** **(A, C)** Fecal propionate levels before the first FMT (pre-FMT) and 6 days after the last FMT (post-FMT) in healthy control (Ctr) recipients which received fecal material from Ctr (A) and EAE (C) donor mice (n = 6). **(B, D)** Fecal butyrate levels pre- and post-FMT in Ctr recipients which received fecal material from Ctr (B) and EAE (D) donor mice (n = 6). Datapoints represent mean ± SEM. Statistical analysis was performed with a Wilcoxon test. Ctr, healthy control; EAE, experimental autoimmune encephalomyelitis; FMT, fecal microbiota transplantation.
